# Trichoderols B-G, Six New Lipids from the Marine Algicolous Fungus *Trichoderma* sp. Z43

**DOI:** 10.3390/md21080453

**Published:** 2023-08-17

**Authors:** Zhen-Zhen Shi, Xiu-Li Yin, Nai-Yun Ji

**Affiliations:** 1 Yantai Institute of Coastal Zone Research, Chinese Academy of Sciences, Yantai 264003, China; zzshi@yic.ac.cn (Z.-Z.S.); xlyin@yic.ac.cn (X.-L.Y.); 2 Shandong Saline-Alkaline Land Modern Agriculture Company, Dongying 257345, China

**Keywords:** *Trichoderma*, lipid, antifungal activity, antimicroalgal activity

## Abstract

Six new lipids, trichoderols B-G (**1**–**6**), along with a known one, triharzianin B (**7**), were isolated from the culture of *Trichoderma* sp. Z43 obtained from the surface of the marine brown alga *Dictyopteris divaricata*. Their structures and relative configurations were identified by interpretation of 1D/2D NMR and MS data. Compounds **1**–**7** were assayed for inhibiting the growth of three phytopathogenic fungi (*Fusarium graminearum*, *Gaeumannomyces graminis*, and *Glomerella cingulata*), four marine phytoplankton species (*Amphidinium carterae*, *Heterocapsa circularisquama*, *Heterosigma akashiwo*, and *Prorocentrum donghaiense*), and one marine zooplankton (*Artemia salina*). Compounds **1**, **4**, and **7** exhibited weak antifungal activities against three phytopathogenic fungi tested with MIC ≥ 64 μg/mL. All compounds displayed moderate antimicroalgal activity with IC_50_ ≥ 15 μg/mL and low toxicity to the brine shrimp *Artemia salina*.

## 1. Introduction

Crop diseases caused by pathogenic fungus have seriously restricted healthy development of agriculture in the world. *Fusarium* head blight of wheat, take-all disease, and plant anthracnose caused by *Fusarium graminearum*, *Gaeumannomyces graminis*, and *Glomerella cingulata*, respectively, lead to huge economic losses in agriculture every year [1,2,3,4,5]. In addition, some marine phytoplankton species including *Amphidinium carterae*, *Heterocapsa circularisquama*, *Heterosigma akashiwo*, and *Prorocentrum donghaiense* can induce red tides that harm aquaculture industry [6,7,8,9]. Thus, it is imperative to search for natural antifungal and antimicroalgal drugs with high activity and safety. On the other hand, *Trichoderma* species are known for producing various metabolites with novel structures and intriguing bioactivities [10,11,12]. In recent years, natural products originated from marine-derived *Trichoderma* species have gained the attention of researchers [13], and a large number of new compounds with significant activities have been found, such as polyketides [14], terpenoids [15,16,17], and steroids [18]. Although *Trichoderma* species have proven to be a treasure-house of new natural products, only a few short-chain lipids have been isolated from this species. For example, triharzianins A-D were purified from *T. harzianum* obtained from mushroom *Tricholoma matsutake* [19], harzianumols A-H were separated from *T. harzianum* obtained from sponge *Petrospongia nigra* [20], and trichoderol A was acquired from *Trichoderma* sp. obtained from soil [21]. Moreover, these lipids displayed multifarious bioactivities such as feeding attractants and antimicrobial activities [19,21]. During our investigation of the chemical diversity and biological activity of marine-derived *Trichoderma*, the epiphytic *Trichoderma* sp. Z43 obtained from the marine brown alga *Dictyopteris divaricata* was examined. Its aerial mycelia grew rapidly. Most of them were white on the PDA medium at 25 °C, and some parts of the plate were yellow. Alternate permutation of the mycelia was observed through a common microscope. As a result, six new lipids, trichoderols B-G (**1**–**6**), along with a known one, triharzianin B (**7**), were isolated and identified. Herein, the details of isolation, structure elucidation, and bioactivities of these compounds are described.

## 2. Results and Discussion

**Figure 1:**
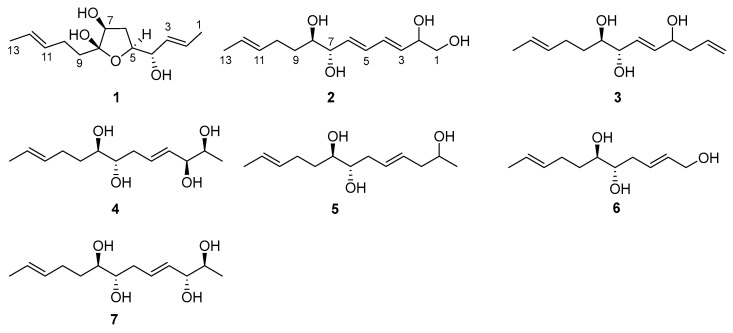
Chemical structures of **1**–**7**.

The organic extracts of marine-derived *Trichoderma* sp. Z43 isolated from the marine brown alga *Dictyopteris divaricata* were subjected to a series of column chromatography processes to produce six new lipids, namely, trichoderols B-G (**1**–**6**), along with a known one, triharzianin B (**7**) (Figure 1). Compound **7** was unambiguously identified by comparing its NMR data and specific rotation value with those reported in the literature [19].

### 2.1. Structural Elucidation

**Figure 2:**
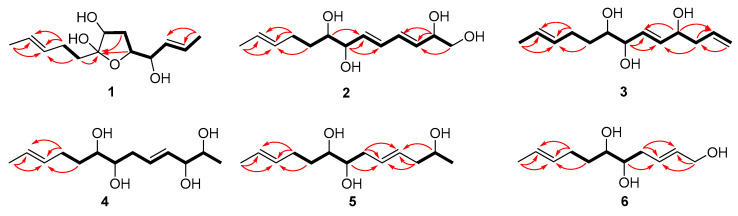
Key COSY and HMBC correlations of **1**–**6** (bold lines for COSY and arrows for HMBC).

**Figure 3:**
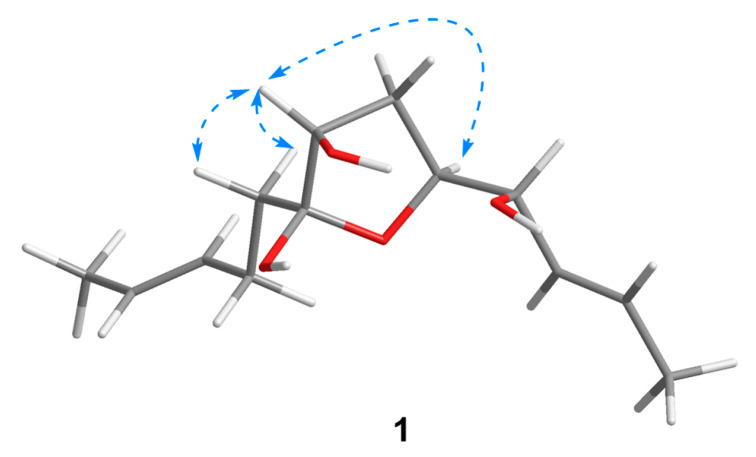
Key NOESY correlations of **1**.

Compound **1** was isolated as a colorless oil with a molecular formula of C_13_H_22_O_4_ established by HRESIMS (*m*/*z* 265.1420 [M + Na]^+^), implying three degrees of unsaturation. The ^1^H NMR spectrum (Table 1) showed two methyl doublets, one double quartet, one double doublet, and two multiplets assignable to four olefinic protons and one double multiplet and two double doublets attributable to three oxymethines. The ^13^C NMR and DEPT spectra (Table 2) displayed the presence of two methyls, three methylenes, seven methines, and one nonprotonated carbon. COSY correlations of H-1/H-2/H-3/H-4/H-5/H-6/H-7 established the linkage of C-1 to C-7 (Figure 2). A pentenyl group was confirmed by the COSY correlation of H-9 with H-10 and the HMBC correlations from H-10/H-13 to C-11 and C-12 and from H-9 to C-11, which was then elongated to C-7 via C-8 by the HMBC correlations from H-6/H-9 to C-8. To satisfy the molecular formula, an ether linkage was situated between C-5 and C-8, which was verified by the HMBC correlation from H-5 to C-8 (Figure 2). Thus, the planar structure of **1** was validated. The double bond at C-2 was attributed to be *trans* by the large coupling constant (*J* = 15.2) between H-2 and H-3. The chemical shifts of other two olefinic carbon atoms (C-11, *δ*_C_ 130.9; C-12, *δ*_C_ 125.5) were highly similar to those of triharzianin B (**7**) (C-11, *δ*_C_ 130.9; C-12, *δ*_C_ 124.6) [19], suggesting a *trans* configuration of the C-11 double bond, which was further verified by the IR absorption at 969 cm^−1^. The relative configurations of H-5, OH-7, and OH-8 were confirmed by NOESY correlations of H-7 with H-5 and H-9 (Figure 3), and the relationship of H-4 and H-5 was deduced to be *threo* due to NMR data that were similar to those of (-)-(*S*)-1-[(2*S*,5*S*)-5-[2-propenyl]tetrahydrofuran-2-yl]prop-2-en-1-ol [22].

Compound **2** was purified as a colorless oil and assigned a molecular formula of C_13_H_22_O_4_ by interpretation of HRESIMS (*m*/*z* 265.1420 [M + Na]^+^) data. In conjunction with HSQC data, the ^1^H NMR spectrum (Table 1) revealed notable signals including one methyl doublet, one double triplet, one double doublet, and one doublet of double doublets ascribable to three oxygenated methines, two double doublets attributable to an oxygenated methylene, and four double doublets and two multiplets assignable to six olefinic protons. DEPT experiments displayed 13 resonances in the ^13^C NMR spectrum, which were assigned to one methyl, three methylenes, and nine methines. COSY correlations of H-1/H-2/H-3/H-4/H-5/H-6/H-7/H-8/H-9/H-10 established the linkage of C-1 to C-10, which was further confirmed by the HMBC correlations from H-1 to C-3, from H-2 to C-3 and C-4, from H-7 to C-5 and C-6, and from H-8 to C-6 (Figure 2). A propenyl group was located at C-10, verified by the HMBC correlations from H-9 to C-11 and from H-10/H-13 to C-11 and C-12 (Figure 2). Thus, the planar structure of **2** was affirmed. The double bonds at C-3 and C-5 were determined to be *trans* by the large coupling constants (*J* = 13.1), and the identical ^13^C NMR data of **2** (C-11, *δ*_C_ 132.2; C-12, *δ*_C_ 126.0) with those of triharzianin C (C-11, *δ*_C_ 132.2; C-12, *δ*_C_ 126.0) [19] suggested the double bond at C-11 to be *trans*. The relationship of OH-7 and OH-8 was assigned to be *erythro* by comparison of NMR data with those for triharzianin C [19]. Although the chemical shifts of C-1, C-2, and C-3 in **2** were the same as those of (*S*)-but-3-ene-1,2-diol [23], the relative configuration of OH-2 could not be confirmed due to the existence of only one chiral carbon atom (C-2) in this moiety. Thus, compound **2** was named (3*E*,5*E*,11*E*)-trideca-3,5,11-trien-1,2,7,8-tetraol, and its structural formula was HOCH_2_CH(OH)CH=CHCH=CHCH(OH)CH(OH)CH_2_CH_2_CH=CHCH_3_.

Compound **3** was acquired as a colorless oil, and its molecular formula was determined to be C_13_H_22_O_3_ by HRESIMS (249.1448 [M + Na]^+^) data. The ^1^H NMR spectrum, along with HSQC data, exhibited notable signals including one methyl doublet, one double triplet, one double doublet, one doublet of double doublets assignable to three oxygenated methines, two broad singlets attributable to a terminal double bond, and three multiplets and two doublets of double doublets ascribable to five olefinic protons. The ^13^C NMR spectrum displayed 13 resonances, sorted into one methyl, four methylenes, and eight methines. The ^1^H and ^13^C NMR data (Table 1 and Table 2) revealed the presence of a similar skeleton to **2**. COSY correlations of H-1/H-2/H-3/H-4/H-5/H-6/H-7/H-8/H-9/H-10/H-11/H-12/H-13 and HMBC correlations from H-1 to C-3, from H-3 to C-2 and C-5, from H-4 to C-2 and C-6, from H-7 to C-5 and C-6, from H-10/H-13 to C-11 and C-12, and from H-9 to C-11 (Figure 2) confirmed the planar structure of **3**. The double bonds at C-5 and C-11 were deduced to be *trans* by the large coupling constant (*J* = 15.5) between H-5 and H-6 and by the identical NMR data of C-11 and C-12 with those of **2**. The relative configurations of OH-7 and OH-8 were determined to be the same as those of **2** on the basis of highly similar chemical shifts and coupling constants of them. Despite the similar NMR data of C-1 to C-5 in **3** with those of 12(*S*)-hydroxy-5(*Z*),8(*Z*),10(*E*),14(*E*)-eicosatetraenoic acid [24], the relative configuration of OH-4 was still unsolved. Compound **3** was named (5*E*,11*E*)-trideca-1,5,11-trien-4,7,8-triol, and its structural formula was CH_2_=CHCH_2_CH(OH)CH=CHCH(OH)CH(OH)CH_2_CH_2_CH=CHCH_3_.

Compound **4** was obtained as a colorless oil and given a molecular formula of C_13_H_24_O_4_ by interpretation of HRESIMS data (*m*/*z* 243.1558 [M]^-^), requiring two degrees of unsaturation. Its NMR data (Table 1 and Table 2) were highly similar to those of triharzianin B (**7**) [19], except for chemical shifts of C-2 and C-3 (C-2, *δ*_C_ 70.2; C-3, *δ*_C_ 76.6 for triharzianin B; C-2, *δ*_C_ 71.6; C-3, *δ*_C_ 77.9 for **4**), which indicated that **4** and triharzianin B possessed the same planar structure. COSY correlations of H-1/H-2/H-3/H-4/H-5/H-6/H-7/H-8/H-9/H-10 and HMBC correlations from H-9 to C-11 and from H-10/H-13 to C-11 and C-12 (Figure 2) further afforded to this structure. The geometry of two double bonds at C-2 and C-11 and the relative configurations of OH-7 and OH-8 were deduced to be the same as those of triharzianin B (**7**) [19] on the basis of their identical NMR data. The relationship of H-2 and H-3 was *threo* by analysis of NMR data with those for separacenes A and B [25]. Therefore, compound **4** was named (4*E*,11*E*)-trideca-4,11-diene-2,3,7,8-tetraol, and its structural formula was CH_3_CH(OH)CH(OH)CH=CHCH_2_CH(OH)CH(OH)CH_2_CH_2_CH=CHCH_3_.

Compound **5** was isolated as a colorless oil. HRESIMS analysis gave the molecular formula of C_13_H_24_O_3_, consistent with two degrees of unsaturation. Its NMR data (Table 1 and Table 2) resembled those of **4**, except for the presence of signals for a methylene and the lack of signals for a hydroxymethine group. COSY correlations of H-1/H-2/H-3 confirmed the kinkage from C-1 to C-3, which was then elongated to C-10 by the HMBC correlations from H-2 to C-4, from H-3/H-6 to C-4 and C-5, and from H-7 to C-5 and the COSY correlations of H-6/H-7/H-8/H-9/H-10. A propenyl group was situated at C-10 by the HMBC correlations from H-9 to C-11 and from H-10/H-13 to C-11 and C-12 (Figure 2). Thus, the planar structure of **5** was confirmed. The configurations of OH-7, OH-8, and the double bond at C-11 were the same as those of **4** due to their similar NMR data, and the geometry of double bond at C-4 was deduced to be *trans* by the large coupling constant (*J* = 15.4) between H-4 and H-5. The relative configuration of OH-2 was uncertain in spite of comparing the NMR data of **5** with those of (*S*)-2-hexanol and (*R*)-octan-2-ol carefully [26]. Thus, compound **5** was named (4*E*,11*E*)-trideca-4,11-dien-2,7,8-triol, and its structural formula was CH_3_CH(OH)CH_2_CH=CHCH_2_CH(OH)CH(OH)CH_2_CH_2_CH=CHCH_3_.

Compound **6** was obtained as a colorless oil and was given a molecular formula of C_11_H_20_O_3_ by analysis of HRESIMS data, requiring two degrees of unsaturation. Its NMR data exhibited high similarities to those of **4** except for the presence of signals for a hydroxymethylene group and lack of signals for a methyl and two hydroxymethine groups. COSY correlations of H-1/H-2/H-3/H-4/H-5/H-6/H-7/H-8 determined the linkage of C-1 to C-8, and a propenyl group was located at C-8, confirmed by the HMBC correlations from H-7 to C-9 and from H-8/H-11 to C-9 and C-10 (Figure 2). Other HMBC correlations further verified the structure of **6** (Figure 2). The large coupling constant (*J* = 15.4) between H-2 and H-3 demonstrated that the double bond at C-2 was *trans*. The identical NMR data of the spin system in **6** (from C-2 to C-11) and **4** (from C-4 to C-13) (Table 1 and Table 2) ascertained the *trans* configuration of a double bond at C-9 and the *erythro* relationship of the vicinal diol (OH-5 and OH-6). Compound **6** was named (2*E*,9*E*)-undeca-2,9-dien-1,5,6-triol, and its structural formula was HOCH_2_CH=CHCH_2_CH(OH)CH(OH)CH_2_CH_2_CH=CHCH_3_.

### 2.2. Bioactivity of Isolated Compounds

Compounds **1**–**7** were assayed for antifungal activity against *Fusarium graminearum*, *Gaeumannomyces graminis*, and *Glomerella cingulata*. The result showed that compounds **1**, **4**, and **7** displayed weak antifungal activity (Table 3). Compound **1** could inhibit the three phytopathogenic fungi tested with MIC values ranging from 64 to 256 μg/mL. Compounds **4** and **7** possessed identical inhibition against *Glomerella cingulata* and *Gaeumannomyces graminis,* with MIC values of 128 and 256 μg/mL, respectively. In addition, all the isolates were evaluated for antimicroalgal activity against *Amphidinium carterae*, *Heterocapsa circularisquama*, *Heterosigma akashiwo*, and *Prorocentrum donghaiense* (Table 4). It was worth noting that only compound **1** was active against all the phytoplankton species tested with IC_50_ values ranging from 15 to 28 μg/mL. Moreover, the antimicroalgal activity of compounds **2**–**7** (IC_50_ ≥ 30 μg/mL) was weaker than that of compound **1**. The above results suggested that the tetrahydrofuran ring could improve antifungal and antimicroalgal activity of these lipids by analysis of their structure–activity relationships. In addition, the brine shrimp lethality of **1**–**7** was also estimated, with the lethal rates against *Artemia salina* of these compounds being less than 10% at 100 μg/mL (Table 4). All isolates showed low toxicity to the brine shrimp *Artermia salina*, which demonstrated the safety of their further exploitation. An in-depth study, such as chemical modification, should be conducted to promote the bioactivity of these compounds, increasing their prospective use in the development of antifungal and antimicroalgal agents.

## 3. Materials and Methods

### 3.1. General Experimental Producers

NMR spectra were obtained on a Bruker Avance III 500 NMR spectrometer (500 and 125 MHz for ^1^H and ^13^C, respectively) using tetramethylsilane (TMS) as an internal standard (Bruker Corp., Billerica, MA, USA). Chemical shifts are reported in parts per million (*δ*) in CDCl_3_/CD_3_OD (*δ*_H_ reported referred to CDCl_3_/CD_3_OD at 7.26/3.31 ppm; *δ*_C_ reported referred to CDCl_3_/CD_3_OD at 77.16/49.00 ppm) and coupling constants (*J*) in Hz. Optical rotations were measured on an SGW-3 polarimeter (Shanghai Shenguang Instrument Co., Ltd., Shanghai, China). IR spectra were recorded on a Nicolet iS50 FT-IR spectrometer (Thermo Fisher Scientific, Waltham, MA, USA); peaks are reported in cm^−1^. High-resolution ESI mass spectra were determined on a Xevo G2-XS QTof mass spectrometer (Water Crop., Milford, MA, USA). Column chromatography (CC) was carried out with silica gel (200–300 mesh, Qingdao Haiyang Chemical Co., Qingdao, China), RP-18 (AAG12S50, YMC Co., Ltd., Kyoto, Japan), and Sephadex LH-20 (GE Healthcare, Uppsala, Sweden). Thin-layer chromatography (TLC) was performed with precoated silica gel plates (GF-254, Qingdao Haiyang Chemical Co., Qingdao, China).

### 3.2. Fungal Material and Fermentation

The fungal strain *Trichoderma* sp. Z43 was isolated from the surface of marine brown alga *Dictyopteris divaricata* collected from Zhoushan, China, in July 2018. The species was identified according to morphological characteristics and analysis of ITS regions of its rDNA, deposited at GenBank (OR196112). Mass fermentation was performed statically at room temperature for 30 days in 200 × 1 L Erlenmeyer flasks, each containing 300 mL of media, by adding 40.0 g glucose, 10.0 g peptone, and 7.0 g yeast extract powder into 1000 mL purified water.

### 3.3. Extraction and Isolation

At the end of fermentation, the mycelia of cultures were obtained by filtration, which were then dried at room temperature, smashed, and extracted with CH_2_Cl_2_ and MeOH (1:1, *v*/*v*). After removing organic solvents under reduced pressure, the residue was partitioned between ethyl acetate (EtOAc) and H_2_O to afford an EtOAc-soluble extract (25.6 g). The filtrate was extracted with EtOAc and then dried to give an extract (10.0 g). The two parts were merged based on the similarity of TLC profiles and subjected to silica gel CC with step-gradient solvent systems of petroleum ether (PE)/EtOAc and CH_2_Cl_2_/MeOH to afford 11 fractions (Frs. 1–11). Fr. 8 eluted with CH_2_Cl_2_/MeOH (20:1) and was further purified by RP-18 CC to afford Fr. 8-1 (MeOH/H_2_O, 3:7), Fr. 8-2 (MeOH/H_2_O, 2:3), and Fr. 8-3 (MeOH/H_2_O, 9:11). Fr. 8-1 was further purified by preparative TLC (CH_2_Cl_2_/MeOH, 20:1) and Sephadex LH-20 CC (MeOH) to give compound **7** (2.6 mg). Compound **1** (4.2 mg) was isolated from Fr. 8-2 by preparative TLC (CH_2_Cl_2_/MeOH, 20:1) and Sephadex LH-20 CC (MeOH). Compounds **3** (4.0 mg), **5** (4.5 mg), and **6** (2.7 mg) were purified from Fr. 8-3 by TLC (CH_2_Cl_2_/MeOH, 10:1) and Sephadex LH-20 CC (MeOH). Fr. 9 eluted with CH_2_Cl_2_/MeOH (10:1) and was further purified by RP-18 CC (MeOH/H_2_O, 1:1) and preparative TLC (CH_2_Cl_2_/MeOH, 10:1) as well as Sephadex LH-20 CC (MeOH) to afford compounds **2** (3.1 mg) and **4** (3.8 mg).

### 3.4. Spectral and Physical Data of Compounds **1**–**6**

Trichoderol B (**1**): colorless oil; [α]^20^_D_ − 10.2 (*c* 0.14, CH_3_OH); IR (KBr) *v*_max_ 3443, 2923, 2854, 1633, 1453, 1384, 969 cm^−1^; ^1^H and ^13^C NMR data, Table 1 and Table 2; HRESIMS *m*/*z* 265.1420 [M + Na]^+^ (calcd for C_13_H_22_NaO_4_, 265.1416).

Trichoderol C (**2**): colorless oil; [α]^20^_D_ − 4.2 (*c* 0.10, CH_3_OH); IR (KBr) *v*_max_ 3430, 2926, 2858, 1633, 1554, 1394, 1030 cm^−1^; ^1^H and ^13^C NMR data, Table 1 and Table 2; HRESIMS *m*/*z* 265.1420 [M + Na]^+^ (calcd for C_13_H_22_NaO_4_, 265.1416).

Trichoderol D (**3**): colorless oil; [α]^20^_D_ + 7.9 (*c* 0.13, CH_3_OH); IR (KBr) *v*_max_ 3422, 2924, 2856, 1661, 1633, 1538, 1394, 1025 cm^−1^; ^1^H and ^13^C NMR data, Table 1 and Table 2; HRESIMS *m/z* 249.1448 [M + Na]^+^ (calcd for C_13_H_22_NaO_3_, 249.1467).

Trichoderol E (**4**): colorless oil; [α]^20^_D_ − 12.2 (*c* 0.13, CH_3_OH); IR (KBr) *v*_max_ 3415, 2924, 1634, 1538, 1385, 1023 cm^−1^; ^1^H and ^13^C NMR data, Table 1 and Table 2; HRESIMS *m*/*z* 243.1588 [M]^−^ (calcd for C_13_H_23_O_4_, 243.1598).

Trichoderol F (**5**): colorless oil; [α]^20^_D_ − 4.1 (*c* 0.15, CH_3_OH); IR (KBr) *v*_max_ 3417, 2924, 2855, 1632, 1554, 1384, 966 cm^−1^; ^1^H and ^13^C NMR data, Table 1 and Table 2; HRESIMS *m*/*z* 251.1614 [M + Na]^+^ (calcd for C_13_H_24_NaO_3_, 251.1623).

Trichoderol G (**6**): colorless oil; [α]^20^_D_ − 5.7 (*c* 0.09, CH_3_OH); IR (KBr) *v*_max_ 3441, 2923, 2853, 1633, 1539, 1385, 985 cm^−1^; ^1^H and ^13^C NMR data, Table 1 and Table 2; HRESIMS *m*/*z* 223.1301 [M + Na]^+^ (calcd for C_11_H_20_NaO_3_, 223.1310).

### 3.5. Assay for Antifungal Activity

Antifungal activity against *Fusarium graminearum*, *Gaeumannomyces graminis*, and *Glomerella cingulata* was performed using the microdilution method in a 96-well plate, as described previously [17]. Briefly, a stock solution of each fungus tested was diluted in potato dextrose broth (PDB) to 5 × 10^5^ cfu/mL. Each sample was prepared in dimethyl sulfoxide (DMSO) and was diluted to final concentrations of 5120, 2560, 1280, 640, 320, 160, 80, 40, 20, 10, and 5 µg/mL in a set of capped test tubes by two-fold serial dilution. An amount of 5 µL diluent was added into each well of a 96-well flat-bottom microtiter plate containing 95 µL fungal suspension (the final sample concentrations were 256 to 0.25 µg/mL), and the fungi were cultivated at 28 °C for 48 h. The MIC value for each sample was defined as the minimum concentration of the compound with invisible microbial growth. Amphotericin B and DMSO were chosen as positive and negative controls, respectively.

### 3.6. Assay for Antimicroalgal Activity

The inhibition of four marine phytoplankton species (*Amphidinium carterae*, *Heterocapsa circularisquama*, *Heterosigma akashiwo*, and *Prorocentrum donghaiense*) was assayed using our previously reported method [17]. In brief, each microalga inoculum was cultured for 5 days using the sterilized f/2 medium in an incubator (20 °C, 14:10 light–dark cycle, 2000 lx light) and reached the exponential growth phase. The microalga suspension was diluted to 4–5 × 10^4^ cells/mL and then added into a 96-well flat-bottom microtiter plate with 195 µL in each well. An amount of 5 µL sample solution (in DMSO) was pipetted into each well (the final sample concentrations were 100 to 0.125 µg/mL) and mixed uniformly. After 24 h inoculation, the live cells were counted using hemocytometer, and the inhibition rate was calculated as follows. Inhibition rate (IR) = (N_CK_ − N_T_)/N_CK_ × 100% (N_CK_: the number of live algal cells under negative control, N_T_: the number of live algal cells under treatment). DMSO and K_2_Cr_2_O_7_ were taken as negative and positive controls, respectively.

### 3.7. Assay for Brine Shrimp Lethal Activity

The inhibition of the brine shrimp *Artermia salina* was assayed according to the procedures described in our previous report [15]. In brief, brine shrimp eggs were left to hatch in sea water for 48 h at 25 °C under natural light. About 10 brine shrimp were placed in a 96-well flat-bottom microtiter plate with a volume of 195 µL sea water in each well. An amount of 5 µL sample solution (in DMSO) was added into each well (the final sample concentrations were 100 to 0.125 µg/mL) and mixed uniformly. The lethality was observed after 24 h of cultivation. DMSO and K_2_Cr_2_O_7_ served as negative and positive controls, respectively.

## 4. Conclusions

Chemical investigation towards the marine algicolous fungus *Trichoderma* sp. Z43 resulted in the isolation of seven lipids, including six new ones (trichoderols B-G (**1**–**6**)) and a known one, triharzianin B (**7**). The C_13_ and C_11_ lipids are rarely found in nature, especially in *Trichoderma* species, and these new compounds greatly enrich the chemical diversity of marine-derived natural products. Finding and stimulating silent biosynthetic gene clusters may be an effective means to excavate this kind of metabolite. These isolations were evaluated for inhibition against three phytopathogenic fungi and four marine phytoplankton species. Several of them exhibited inhibition of one or more fungi/plankton species tested, and the tetrahydrofuran ring could improve antifungal and antimicroalgal activity of these lipids by analysis of their structure–activity relationships. Moreover, all isolates exhibited low toxicity to the brine shrimp *Artermia salina*, suggesting the security for their further exploitation.

## Figures and Tables

**Table 1:** ^1^H NMR data for **1**–**6** (δ in ppm, *J* in Hz).

Pos	1 (CDCl_3_)	2 (CD_3_OD)	3 (CDCl_3_)	4 (CD_3_OD)	5 (CD_3_OD)	6 (CD_3_OD)
**1a**	1.72, d (6.6)	3.48, dd (11.1, 5.1)	5.17, brs	1.14, d (6.4)	1.14, d (6.2)	4.02, d (5.2)
**1b**		3.46, dd (11.2, 6.8)	5.14, brs			
**2**	5.81, dq (15.2, 6.6)	4.15, dt (6.3, 5.2)	5.80, m	3.65, qd (6.4, 6.1)	3.75, sext (6.2)	5.69, dt (15.4, 5.2)
**3a**	5.28, dd (15.2, 7.4)	5.71, dd (13.1, 6.3)	2.37, dddt (13.9, 6.6, 5.3, 1.2)	3.84, dd (7.1, 6.1)	2.18, m	5.72, dt (15.4, 6.7)
**3b**			2.28, dddt (13.9, 7.4, 6.6, 1.0)		2.14, m	
**4a**	4.20, dm (7.4)	6.32, dd (13.1, 10.6)	4.24, dt (6.1, 5.5)	5.60, ddm (15.5, 7.1)	5.54, dt (15.4, 6.4)	2.31, m
**4b**						2.19, m
**5**	4.52 dd (5.5, 3.1)	6.29, dd (13.1, 10.6)	5.84, ddd (15.5, 5.6, 0.9)	5.75, m	5.56, dt (15.4, 6.4)	3.44, m
**6a**	2.44, dd (13.5, 6.9)	5.73, dd (13.1, 5.7)	5.73, ddd (15.5, 6.3, 1.1)	2.33, m	2.35, dm (13.8)	3.43, m
**6b**	1.45, dddd (13.5, 5.5, 2.6, 1.2)			2.22, dd (14.2, 7.3)	2.12, m	
**7**	3.88, dd (6.9, 2.6)	3.93, dd (6.5, 5.7)	3.96, dd (6.3, 5.9)	3.47, m	3.40, m	1.52, m
**8a**		3.42, ddd (9.4, 5.7, 3.2)	3.49, ddd (9.3, 5.9, 3.5)	3.44, m	3.39, m	2.15, m
**8b**						2.04, m
**9a**	1.99, m	1.54, m	1.58, m	1.53, m	1.67, m	5.45, m
**9b**		1.38, m	1.49, m		1.41, m	
**10a**	2.24, m	2.16, m	2.18, m	2.16, m	2.19, m	5.45, m
**10b**		2.03, m	2.09, m	2.04, m	2.02, m	
**11**	5.49, m	5.44, m	5.44, m	5.46, m	5.46, m	1.64, d (4.7)
**12**	5.50, m	5.43, m	5.47, m	5.46, m	5.46, m	
**13**	1.64, d (4.6)	1.63, d (5.2)	1.65, d (6.0)	1.64, d (3.9)	1.64, d (3.9)	

**Table 2:** ^13^C NMR data for **1**–**6** (δ in ppm).

Pos	1 (CDCl_3_)	2 (CD_3_OD)	3 (CDCl_3_)	4 (CD_3_OD)	5 (CD_3_OD)	6 (CD_3_OD)
**1**	18.1, CH_3_	67.2, CH_2_	118.9, CH_2_	18.7, CH_3_	22.9, CH_3_	63.7, CH_2_
**2**	131.4, CH	73.9, CH	134.0, CH	71.6, CH	68.5, CH	132.7, CH
**3**	126.5, CH	134.3, CH	42.0, CH_2_	77.9, CH	43.6, CH_2_	129.9, CH
**4**	80.0, CH	132.2, CH	70.8, CH	132.9, CH	130.1, CH	37.1, CH_2_
**5**	78.2, CH	132.6, CH	135.4, CH	131.1, CH	130.7, CH	75.0, CH
**6**	36.1, CH_2_	134.3, CH	130.0, CH	37.3, CH_2_	37.3, CH_2_	73.9, CH
**7**	74.2, CH	76.7, CH	75.4, CH	75.0, CH	75.9, CH	33.9, CH_2_
**8**	111.3, C	75.1, CH	74.2, CH	73.9, CH	74.7, CH	30.0, CH_2_
**9**	28.5, CH_2_	33.7, CH_2_	32.8, CH_2_	33.9, CH_2_	33.6, CH_2_	132.2, CH
**10**	26.5, CH_2_	29.9, CH_2_	28.9, CH_2_	30.0, CH_2_	29.9, CH_2_	126.0, CH
**11**	130.9, CH	132.2, CH	130.7, CH	132.2, CH	132.3, CH	18.1, CH_3_
**12**	125.5, CH	126.0, CH	125.9, CH	126.0, CH	126.0, CH	
**13**	18.1, CH_3_	18.1, CH_3_	18.1, CH_3_	18.1, CH_3_	18.1, CH_3_	

**Table 3:** Inhibition of three phytopathogenic fungi by **1**–**7**.

Compounds	MIC (μg/mL)		
	*Fusarium* *graminearum*	*Gaeumannomyces graminis*	*Glomerella cingulata*
**1**	256	256	64
**2**	-	-	-
**3**	-	-	-
**4**	-	256	128
**5**	-	-	-
**6**	-	-	-
**7**	-	256	128
amphotericin B	2.0	2.0	1.0

-: no inhibition effect at 256 μg/mL.

**Table 4:** Inhibition of phytoplankton species by **1**–**7**.

Compounds	IC_50_ (μg/mL)				Lethal Rate (at 100 μg/mL)
	*Amphidinium carterae*	*Heterocapsa circularisquama*	*Heterosigma akashiwo*	*Prorocentrum donghaiense*	*Artemia salina*
**1**	15	24	28	22	9.6%
**2**	61	-	43	37	5.2%
**3**	66	78	-	-	4.6%
**4**	-	-	53	42	6.1%
**5**	50	68	-	-	8.4%
**6**	44	-	82	-	3.2%
**7**	-	-	49	30	5.4%
K_2_Cr_2_O_7_	1.2	1.0	0.8	1.4	100%

-: no inhibition effect at 100 μg/mL.

## Data Availability

Data of the compounds are available in Supplementary Materials.

## Supplementary Materials

The following supporting information can be downloaded at: https://www.mdpi.com/article/10.3390/md21080453/s1, Figures S1–S42: 1D/2D NMR, HRESIMS spectra of **1**–**6**.
