# Supplementary material for: Trichoderols B-G, Six New Lipids from the Marine Algicolous Fungus Trichoderma sp. Z43
Source: Mar Drugs. 2023 Aug 17;21(8):453. doi: 10.3390/md21080453 (PMC10456296; doi:10.3390/md21080453)
Supplement: Supplementary file 1 [file marinedrugs-21-00453-s001.zip › Supporting Information.pdf]

Supplementary Materials

Trichoderols B-G, Six New Lipids from the Marine Algicolous Fungus *Trichoderma* sp. Z43

Zhen-Zhen Shi[1], Xiu-Li Yin[1], Nai-Yun Ji[1, 2]*

1 Yantai Institute of Coastal Zone Research, Chinese Academy of Sciences, Yantai 264003, China; zzshi@yic.ac.cn (Z.-Z.S.); xlyin@yic.ac.cn (X.-L.Y.)

2 Shandong Saline-Alkaline Land Modern Agriculture Company, Dongying 257345, China

* Correspondence: nyji@yic.ac.cn; Tel.: +86-535-210-9176

Table of Contents

Figure S1. ^{1}H NMR spectrum of compound **1** in $CDCl_3$
Figure S2. ^{13}C NMR and DEPT spectra of compound **1** in $CDCl_3$
Figure S3. HSQC spectrum of compound **1** in $CDCl_3$
Figure S4. HMBC spectrum of compound **1** in $CDCl_3$
Figure S5. COSY spectrum of compound **1** in $CDCl_3$
Figure S6. NOESY spectrum of compound **1** in $CDCl_3$
Figure S7. HRESIMS spectrum of compound **1**
Figure S8. ^{1}H NMR spectrum of compound **2** in CD_3OD
Figure S9. ^{13}C NMR and DEPT spectra of compound **2** in CD_3OD
Figure S10. HSQC spectrum of compound **2** in CD_3OD
Figure S11. HMBC spectrum of compound **2** in CD_3OD
Figure S12. COSY spectrum of compound **2** in CD_3OD
Figure S13. NOESY spectrum of compound **2** in CD_3OD
Figure S14. HRESIMS spectrum of compound **2**
Figure S15. ^{1}H NMR spectrum of compound **3** in $CDCl_3$
Figure S16. ^{13}C NMR and DEPT spectra of compound **3** in $CDCl_3$
Figure S17. HSQC spectrum of compound **3** in $CDCl_3$
Figure S18. HMBC spectrum of compound **3** in $CDCl_3$
Figure S19. COSY spectrum of compound **3** in $CDCl_3$
Figure S20. NOESY spectrum of compound **3** in $CDCl_3$
Figure S21. HREIMS spectrum of compound **3**
Figure S22. ^{1}H NMR spectrum of compound **4** in CD_3OD
Figure S23. ^{13}C NMR and DEPT spectra of compound **4** in CD_3OD
Figure S24. HSQC spectrum of compound **4** in CD_3OD
Figure S25. HMBC spectrum of compound **4** in CD_3OD
Figure S26. COSY spectrum of compound **4** in CD_3OD
Figure S27. NOESY spectrum of compound **4** in CD_3OD
Figure S28. HRESIMS spectrum of compound **4**
Figure S29. ^{1}H NMR spectrum of compound **5** in CD_3OD
Figure S30. ^{13}C NMR and DEPT spectra of compound **5** in CD_3OD
Figure S31. HSQC spectrum of compound **5** in CD_3OD
Figure S32. HMBC spectrum of compound **5** in CD_3OD
Figure S33. COSY spectrum of compound **5** in CD_3OD
Figure S34. NOESY spectrum of compound **5** in CD_3OD
Figure S35. HRESIMS spectrum of compound **5**
Figure S36. ^{1}H NMR spectrum of compound **6** in CD_3OD
Figure S37. ^{13}C NMR and DEPT spectra of compound **6** in CD_3OD
Figure S38. HSQC spectrum of compound **6** in CD_3OD

Figure S39. HMBC spectrum of compound **6** in CD_3OD
Figure S40. COSY spectrum of compound **6** in CD_3OD
Figure S41. NOESY spectrum of compound **6** in CD_3OD
Figure S42. HRESIMS spectrum of compound **6**

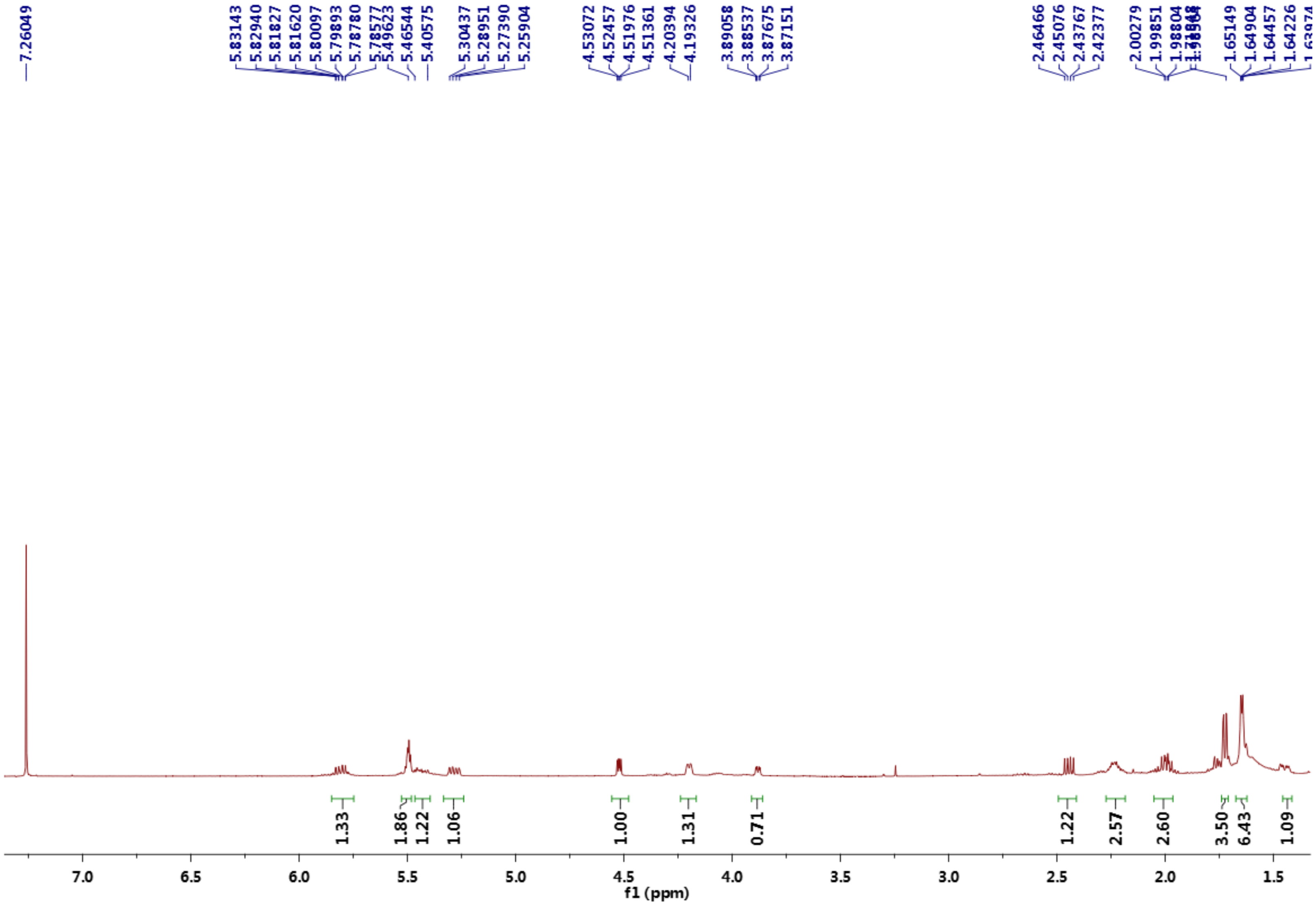

Figure S1. ^{1}H NMR spectrum of compound **1** in $CDCl_3$

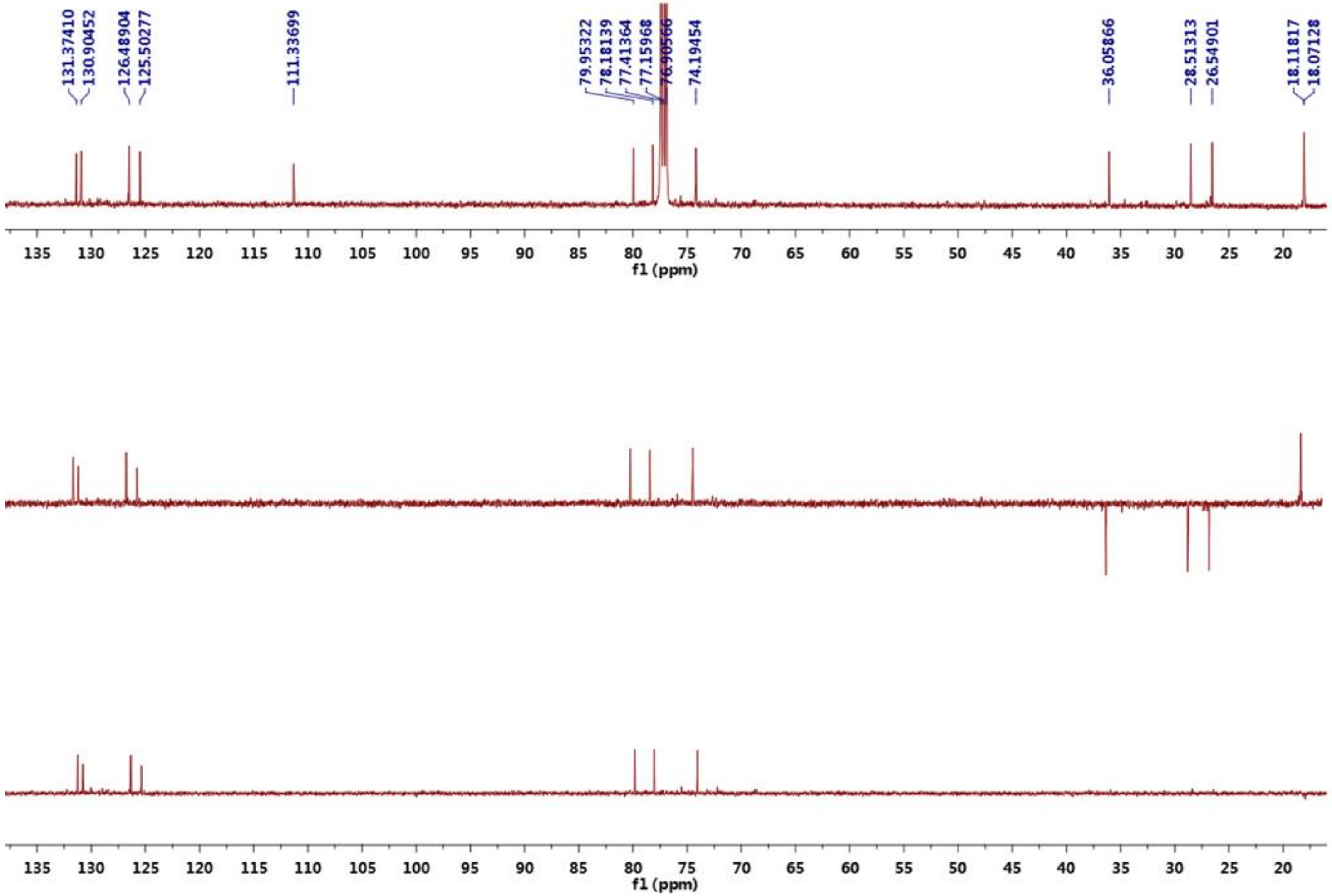

Figure S2. ^{13}C NMR and DEPT spectra of compound **1** in $CDCl_3$

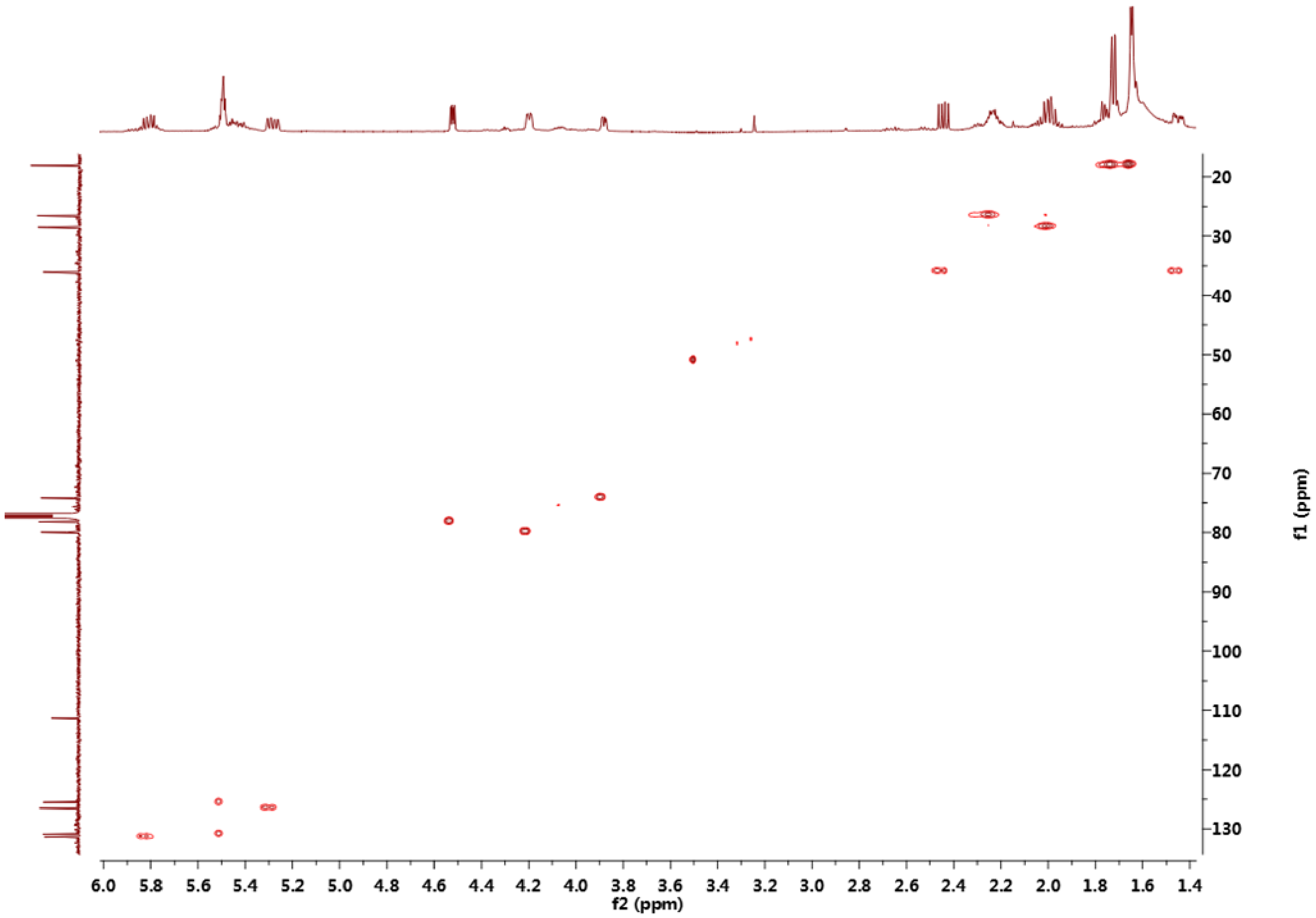

Figure S3. HSQC spectrum of compound **1** in $CDCl_3$

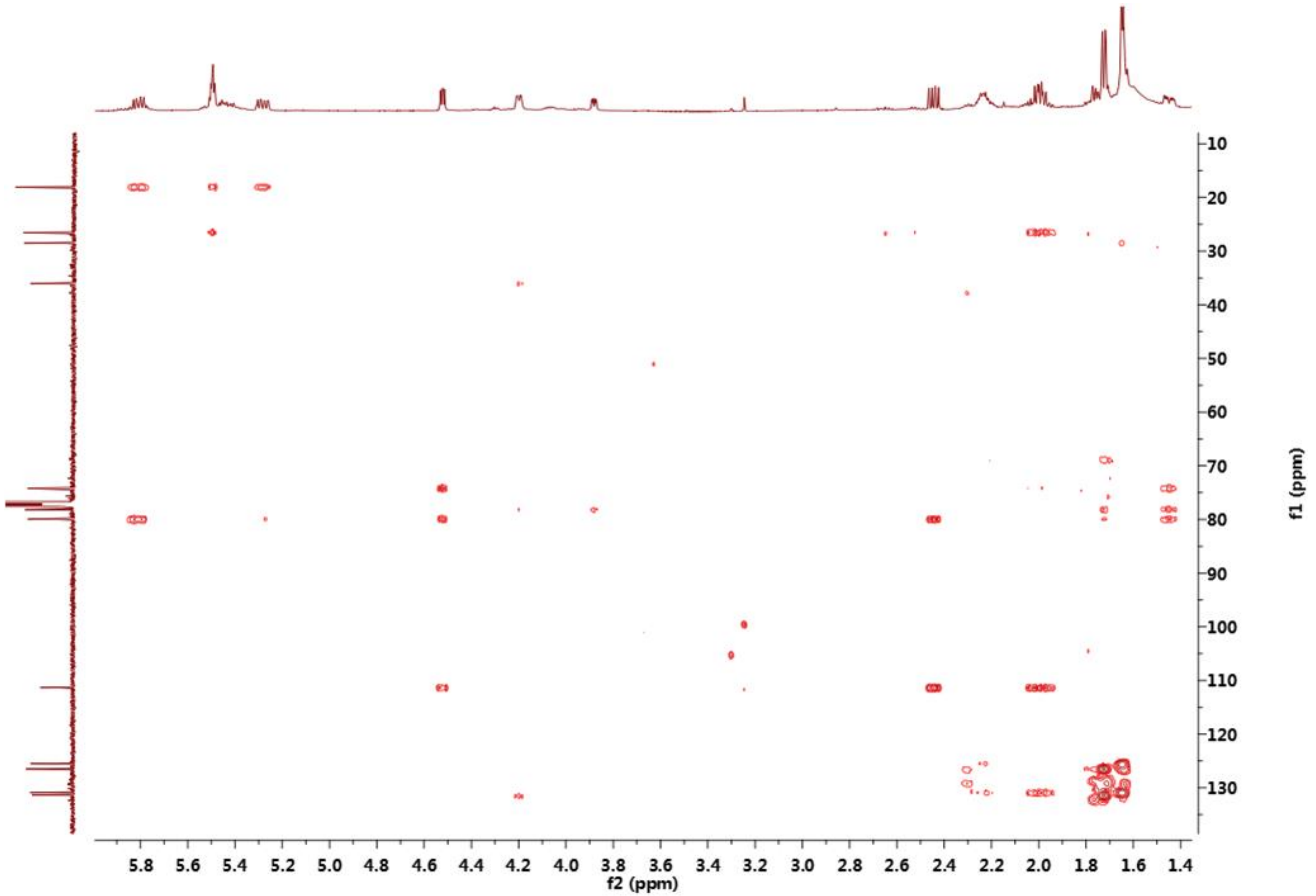

Figure S4. HMBC spectrum of compound **1** in $CDCl_3$

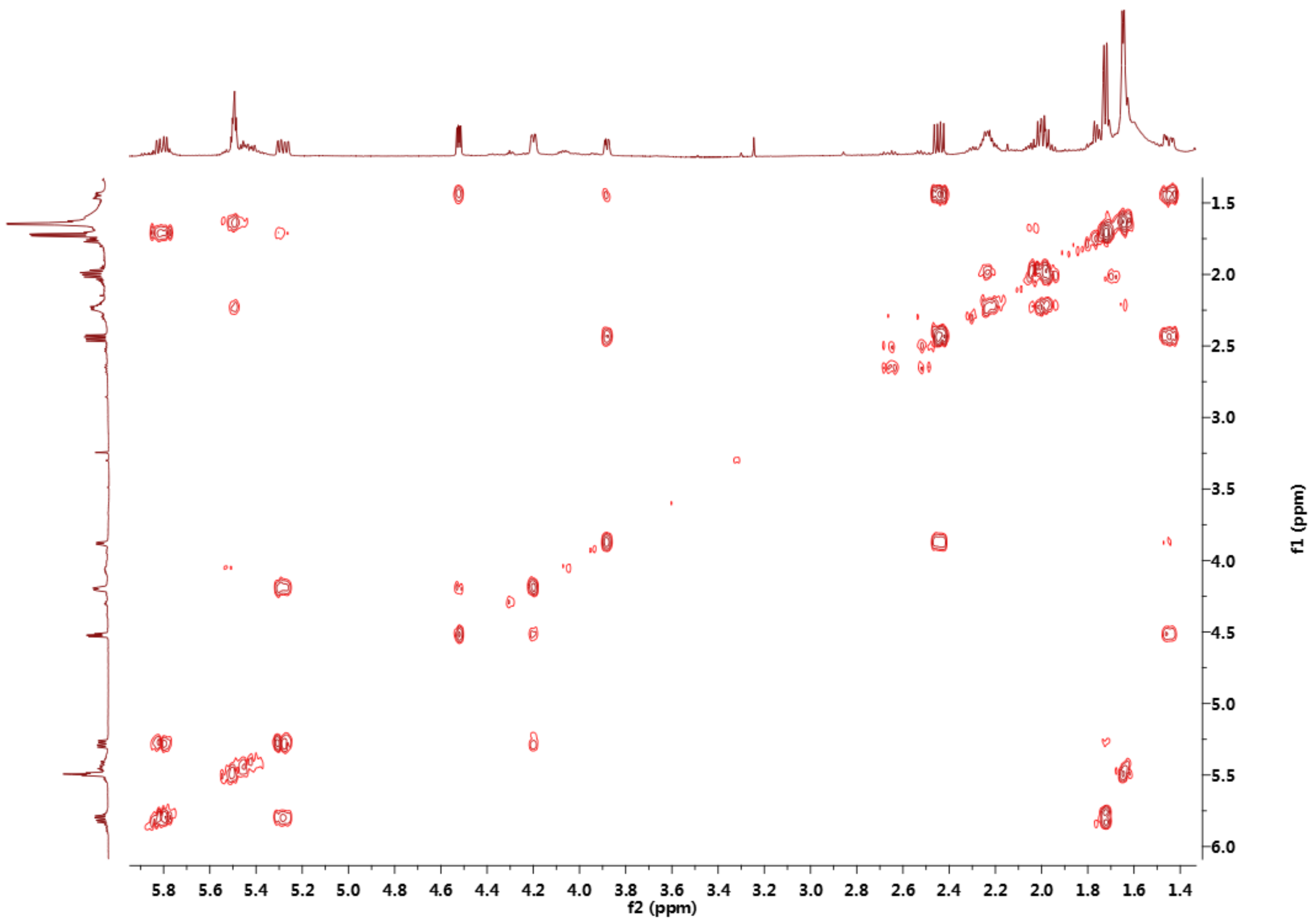

Figure S5. COSY spectrum of compound **1** in $CDCl_3$

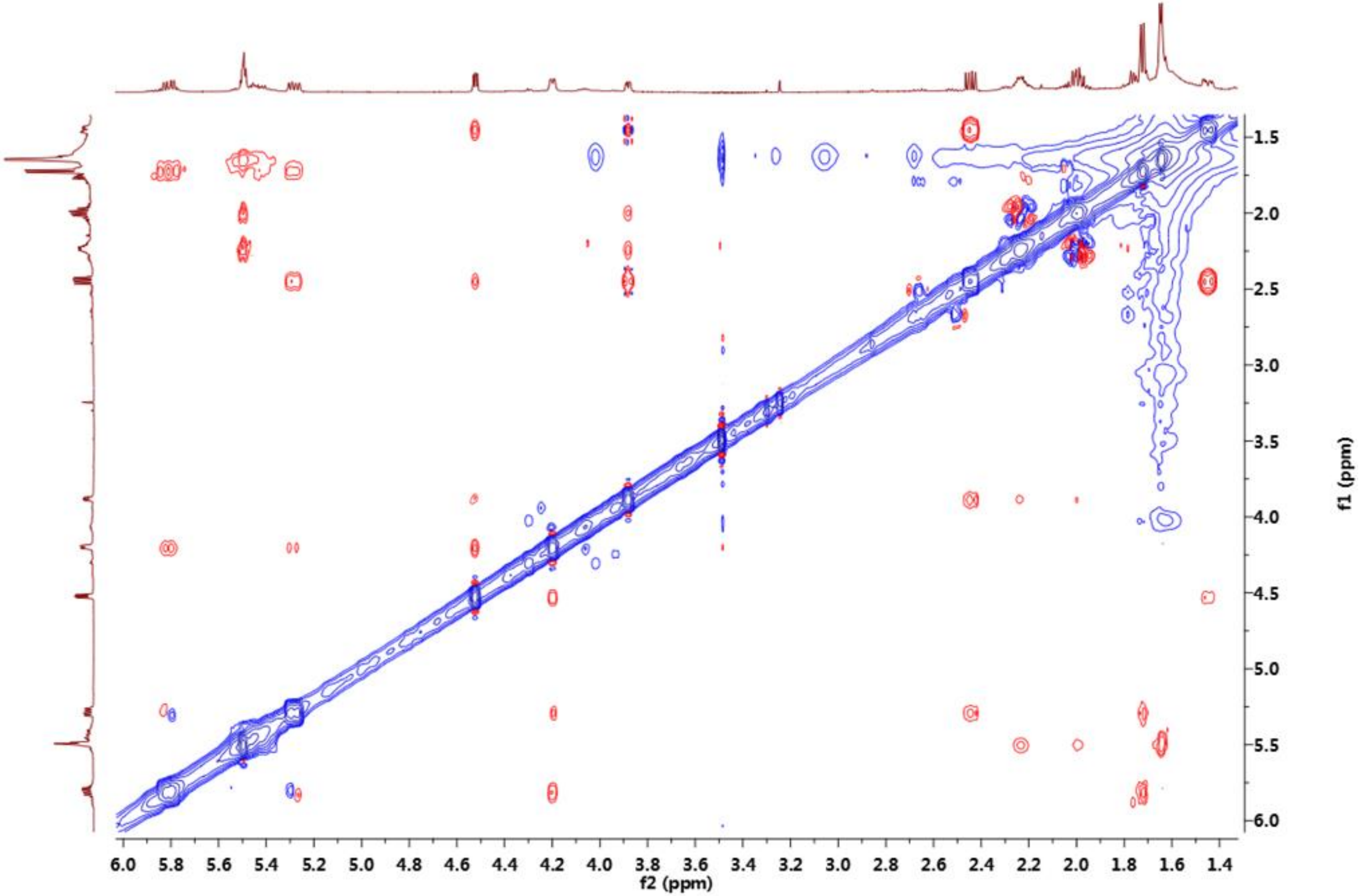

Figure S6. NOESY spectrum of compound **1** in $CDCl_3$

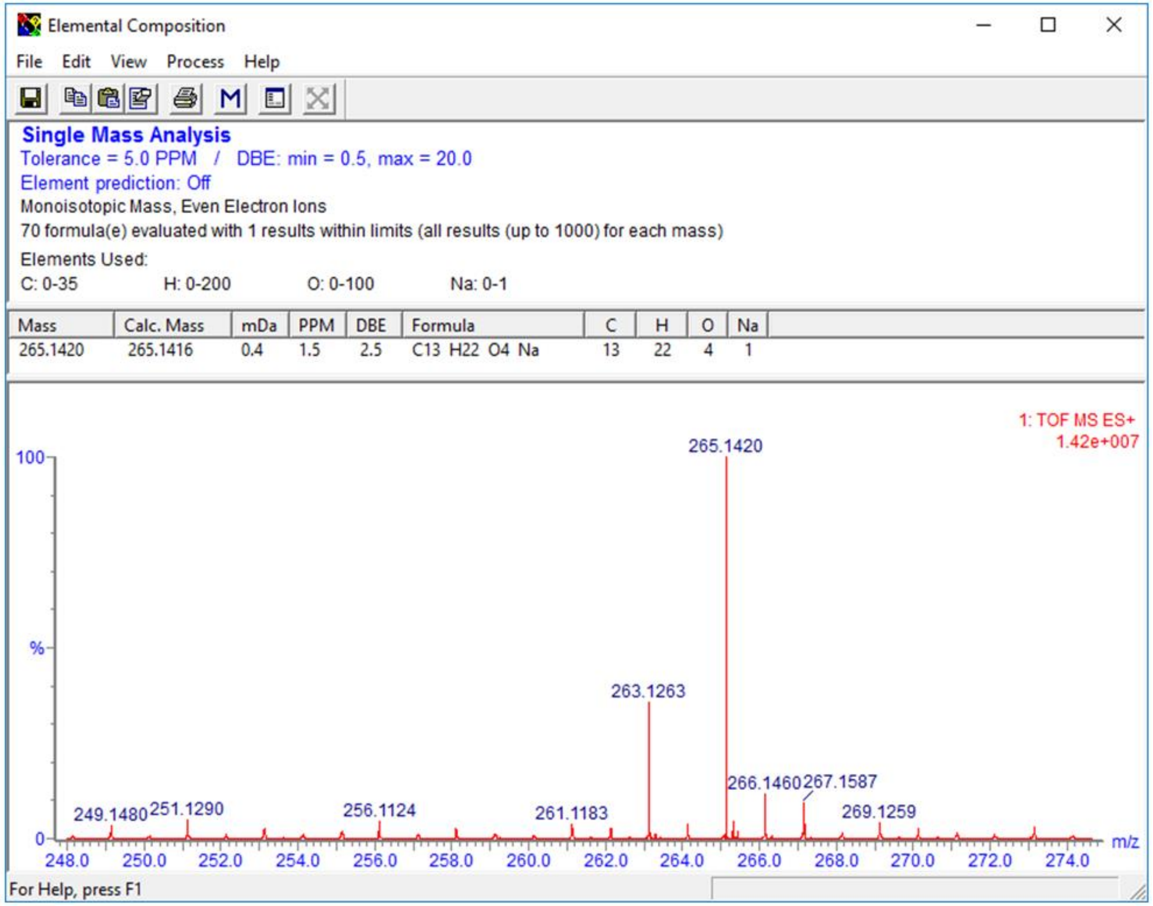

Figure S7. HRESIMS spectrum of compound **1**

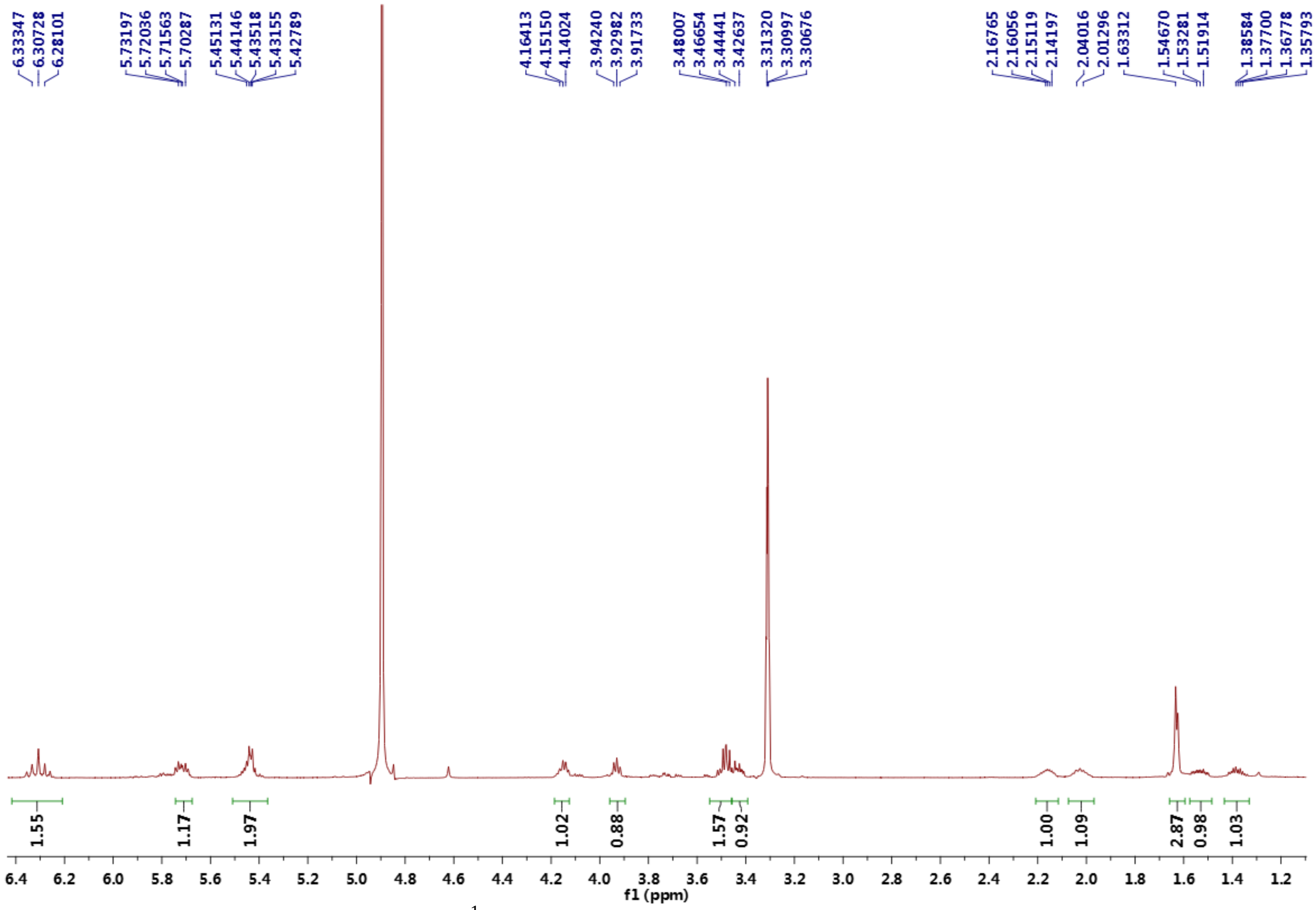

Figure S8. ^{1}H NMR spectrum of compound **2** in CD_3OD

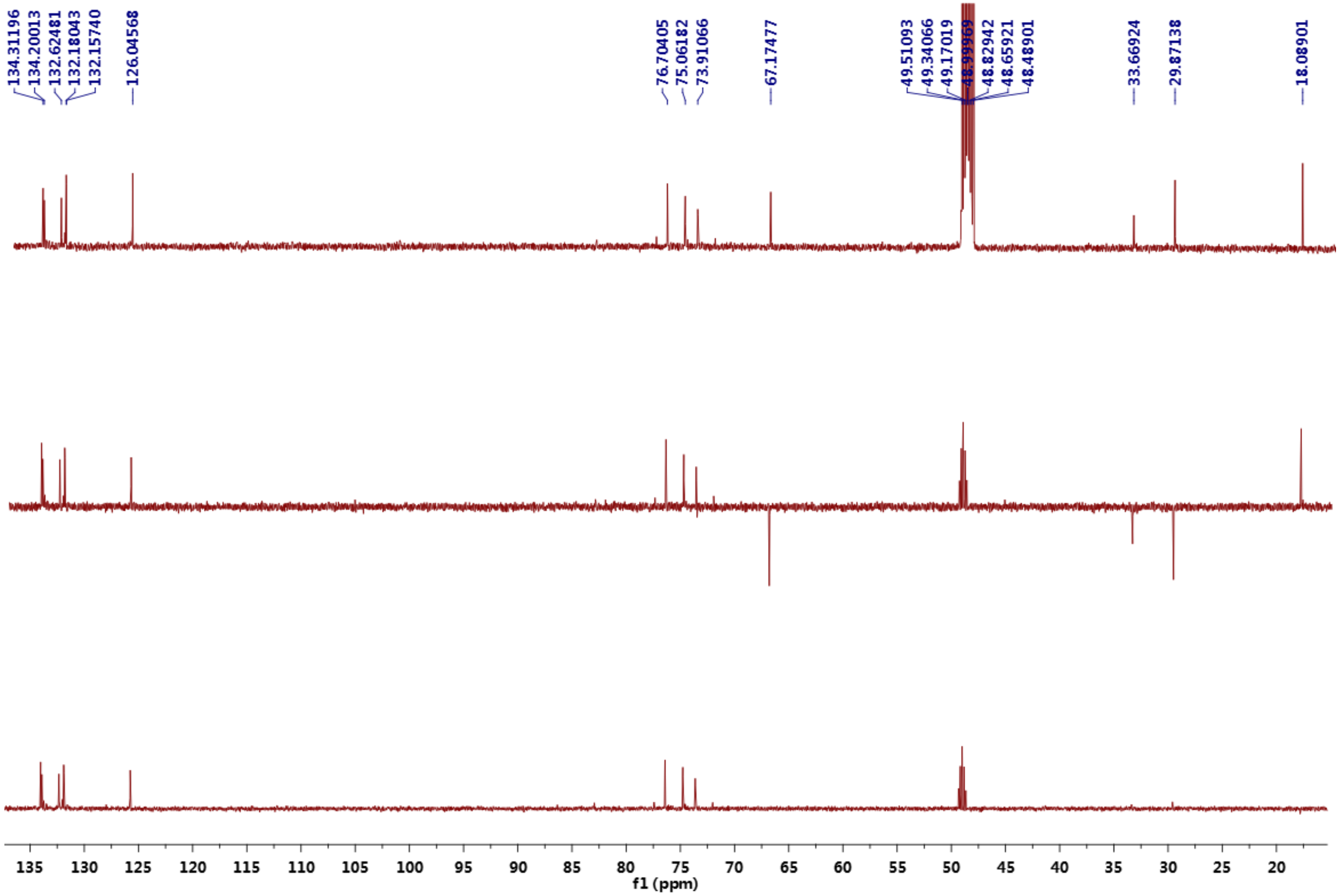

Figure S9. ^{13}C NMR and DEPT spectra of compound **2** in CD_3OD

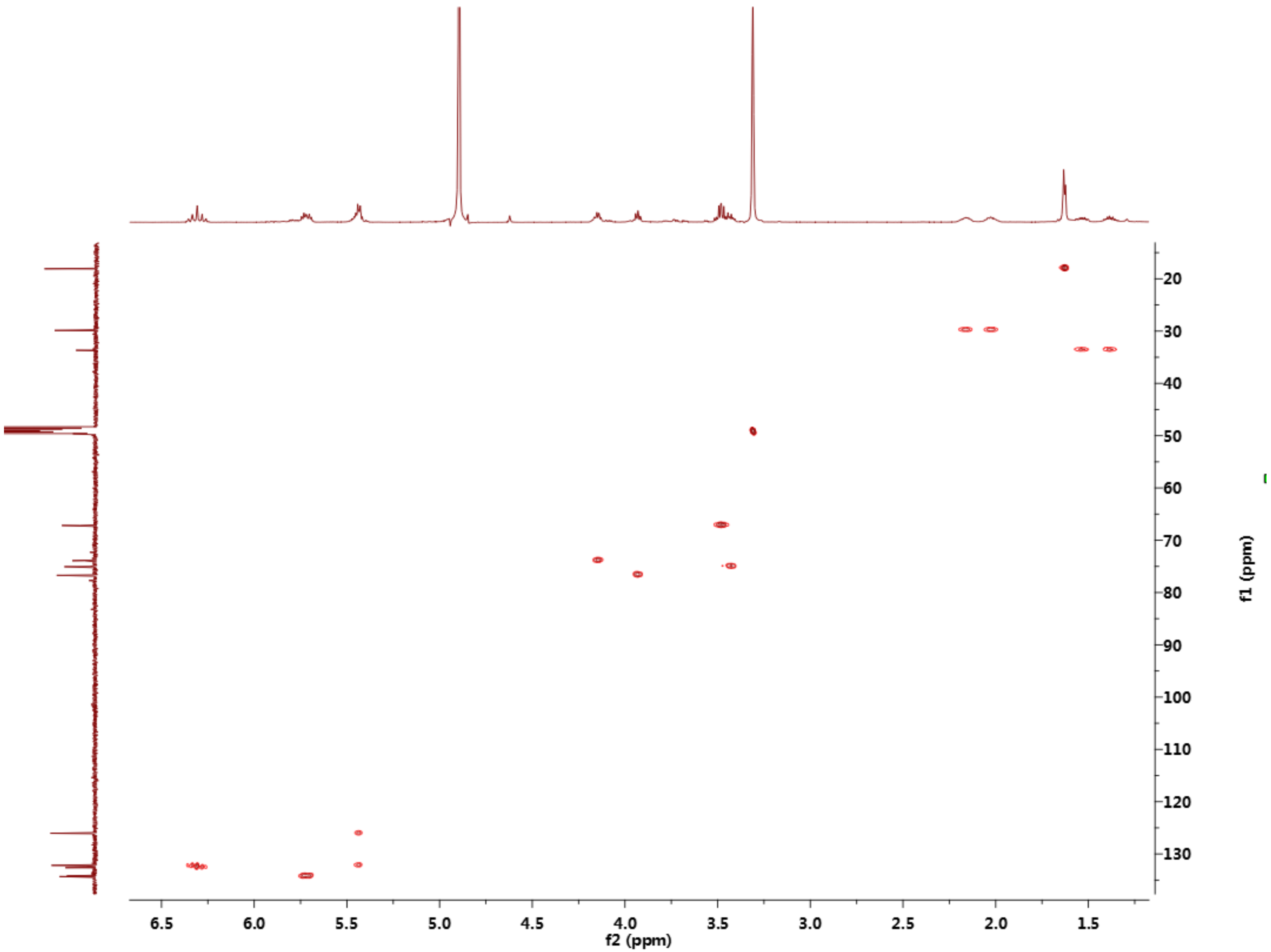

Figure S10. HSQC spectrum of compound **2** in CD_3OD

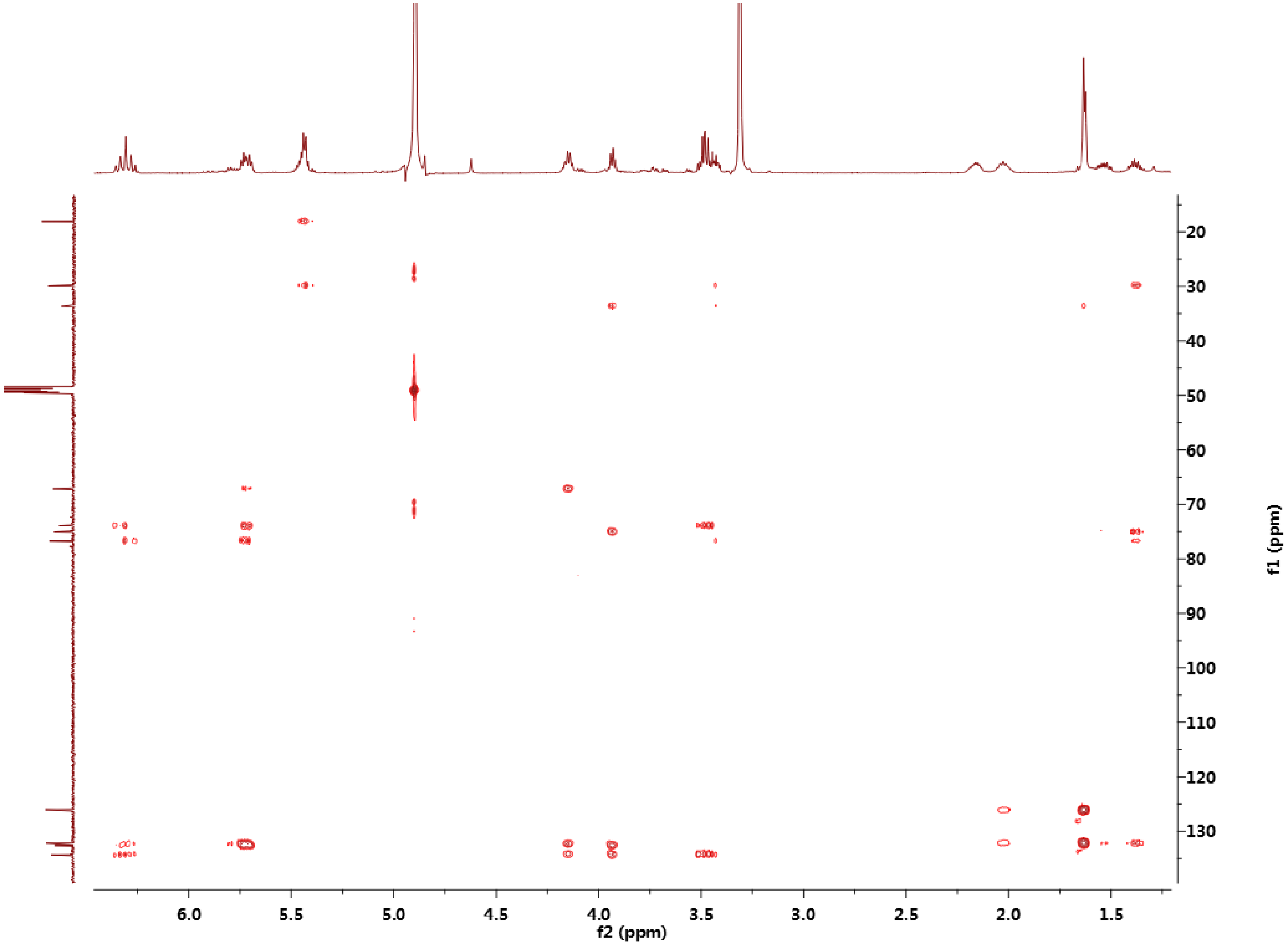

Figure S11. HMBC spectrum of compound **2** in CD_3OD

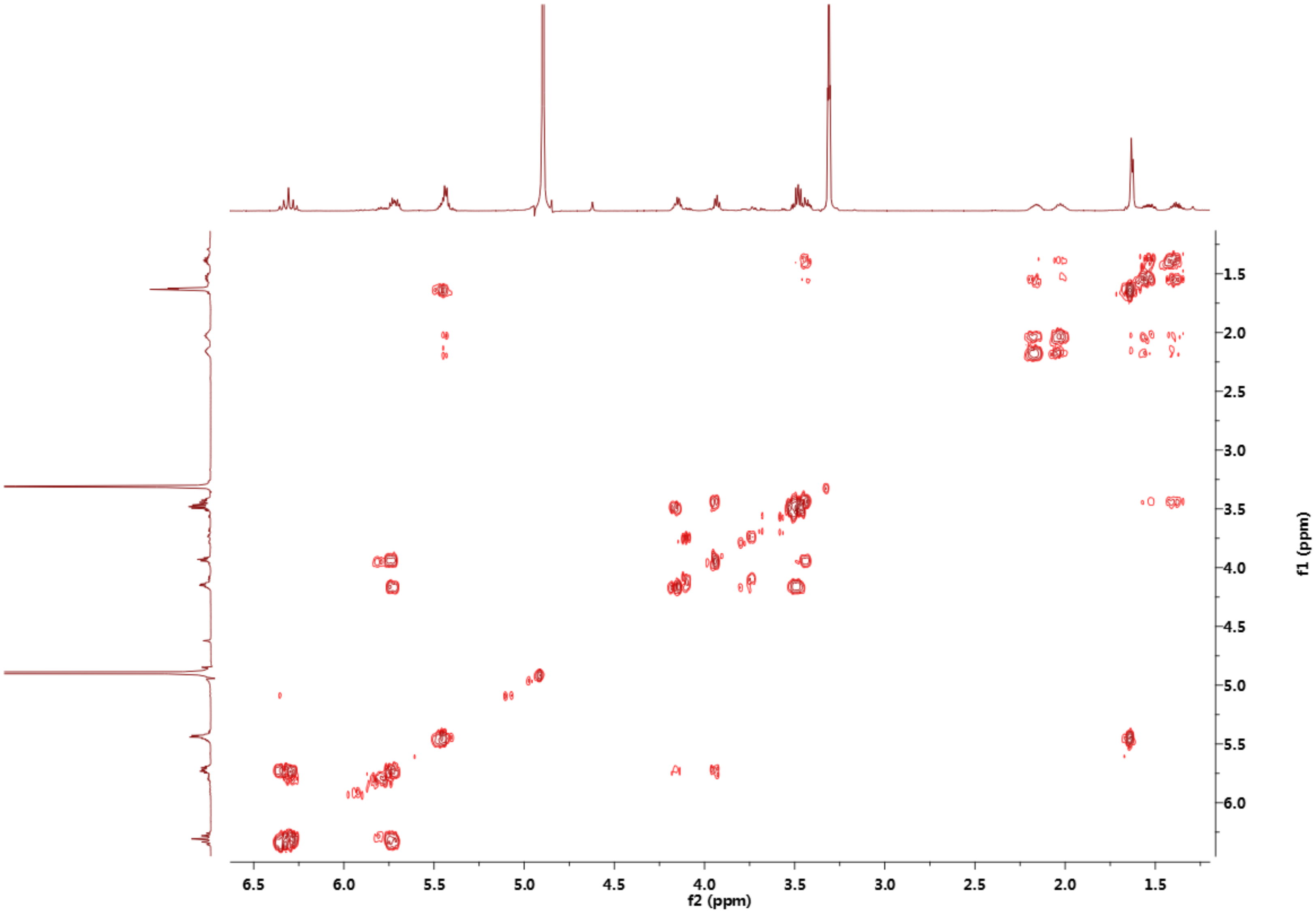

Figure S12. COSY spectrum of compound **2** in CD_3OD

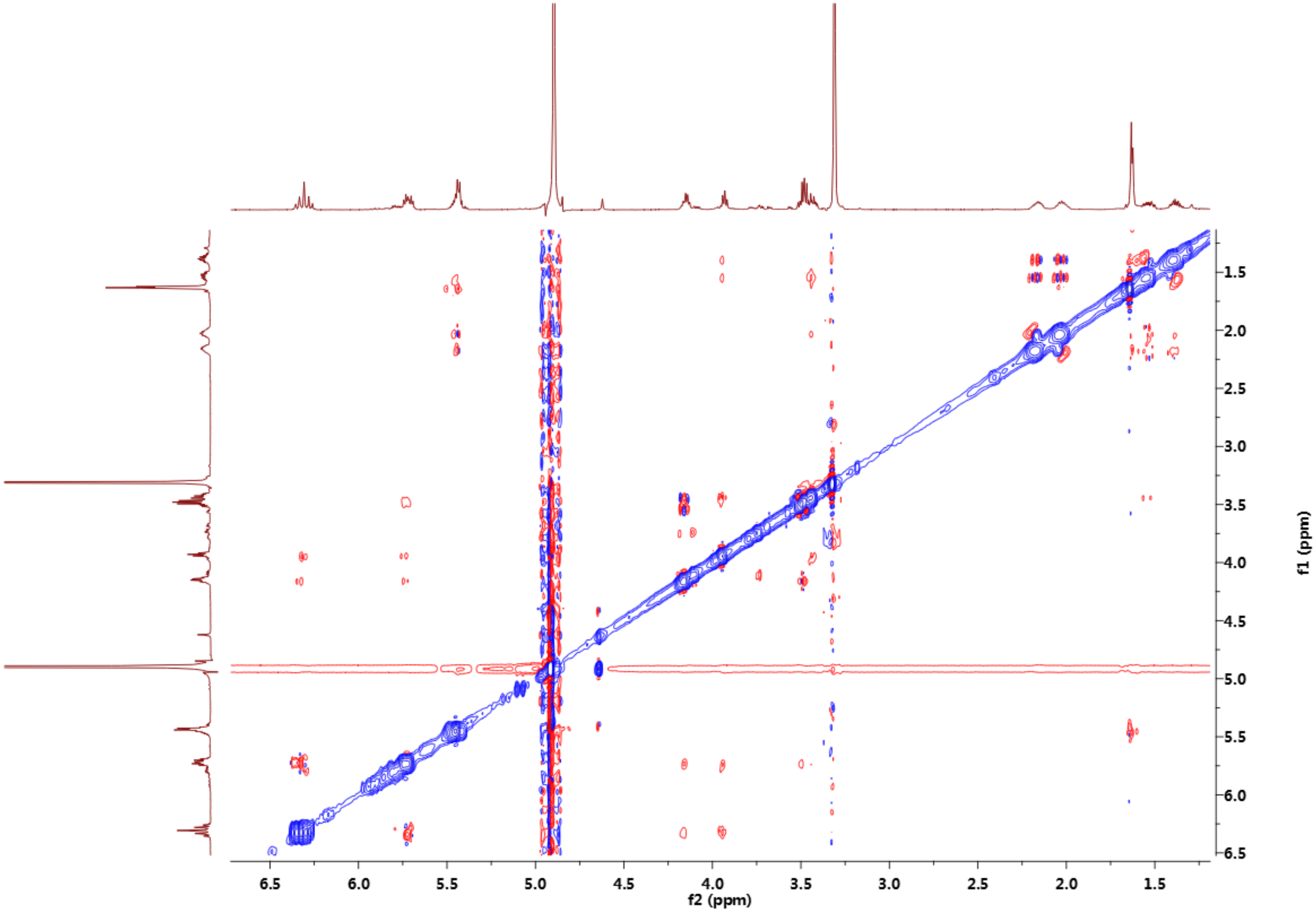

Figure S13. NOESY spectrum of compound **2** in CD_3OD

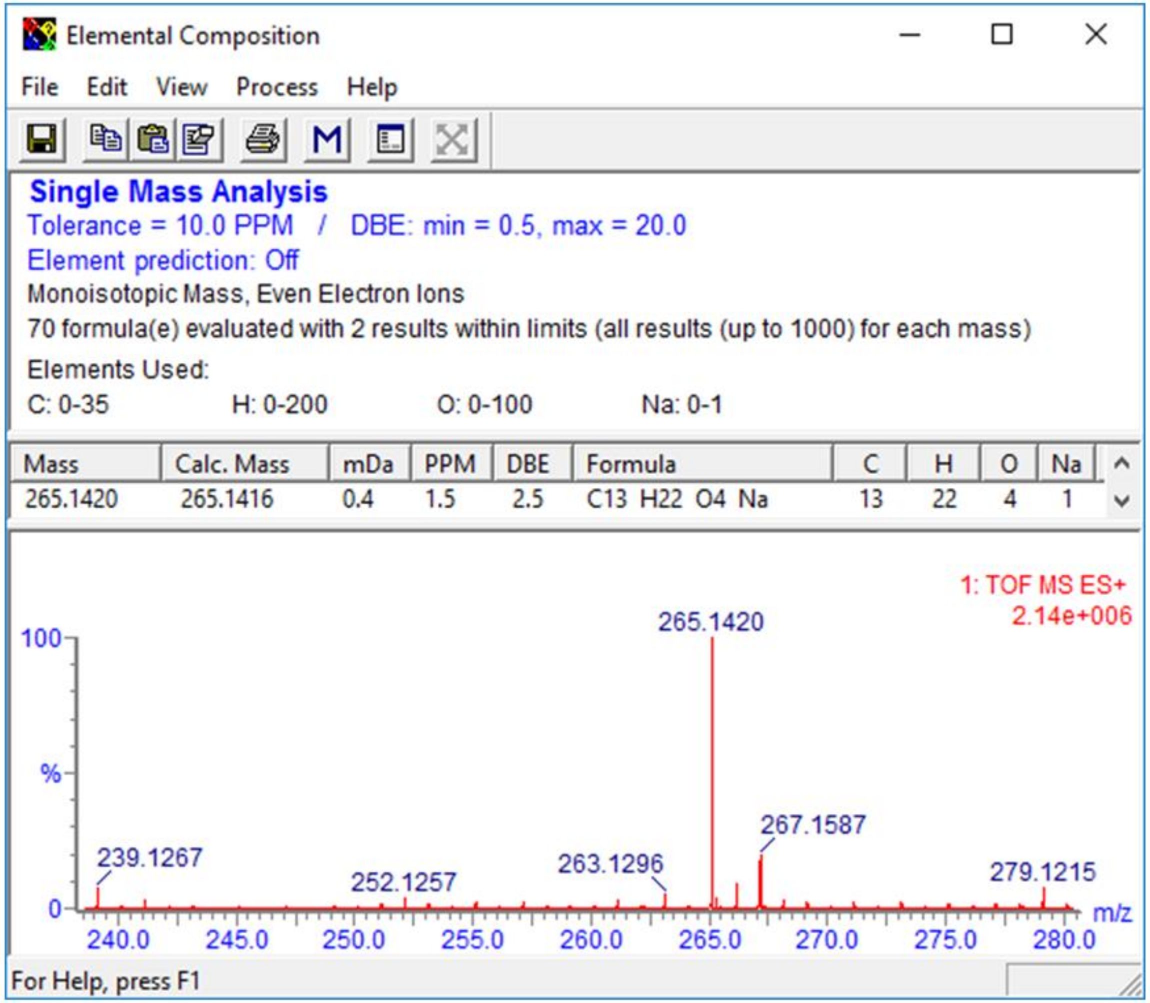

Figure S14. HRESIMS spectrum of compound **2**

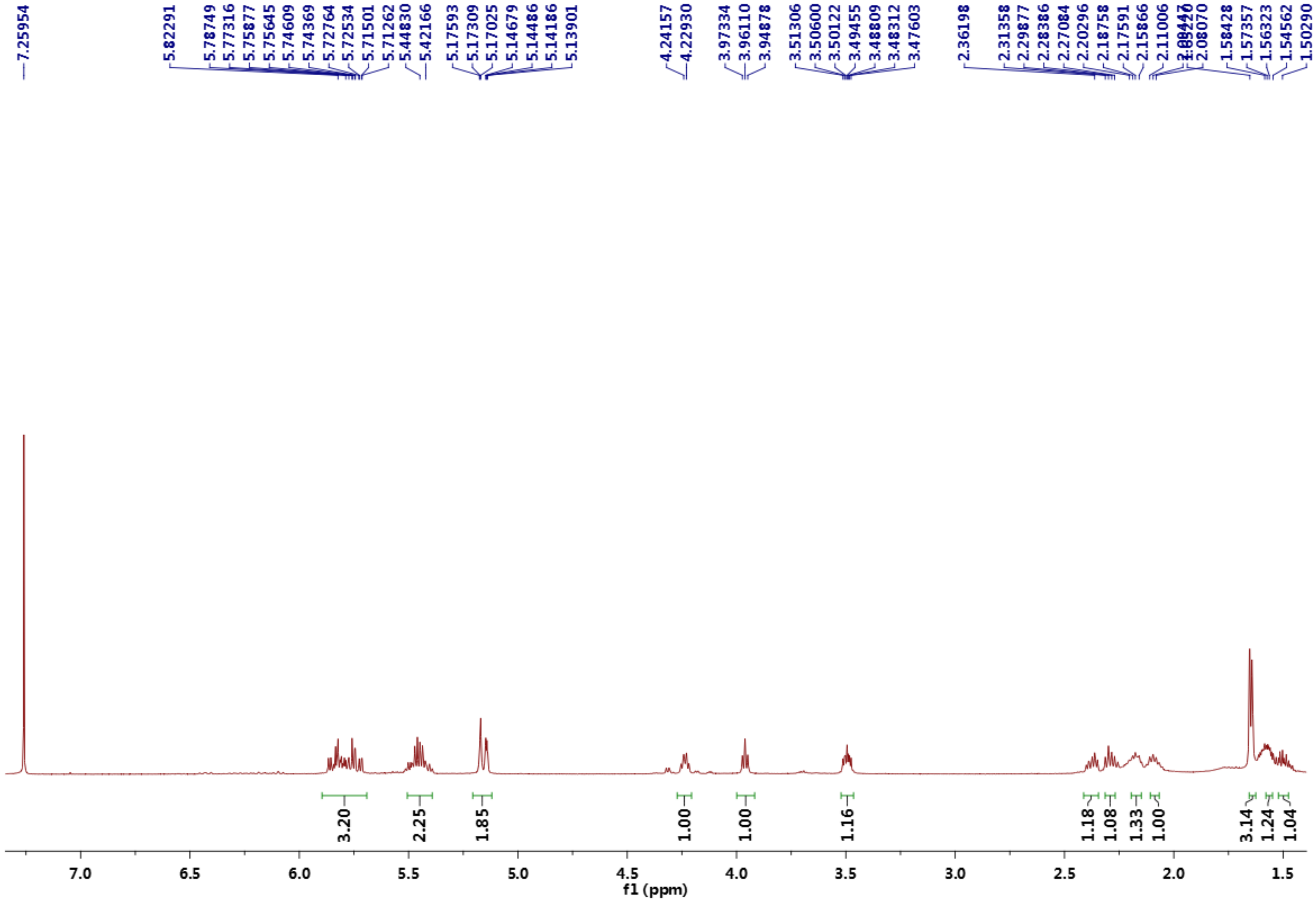

Figure S15. ^{1}H NMR spectrum of compound **3** in $CDCl_3$

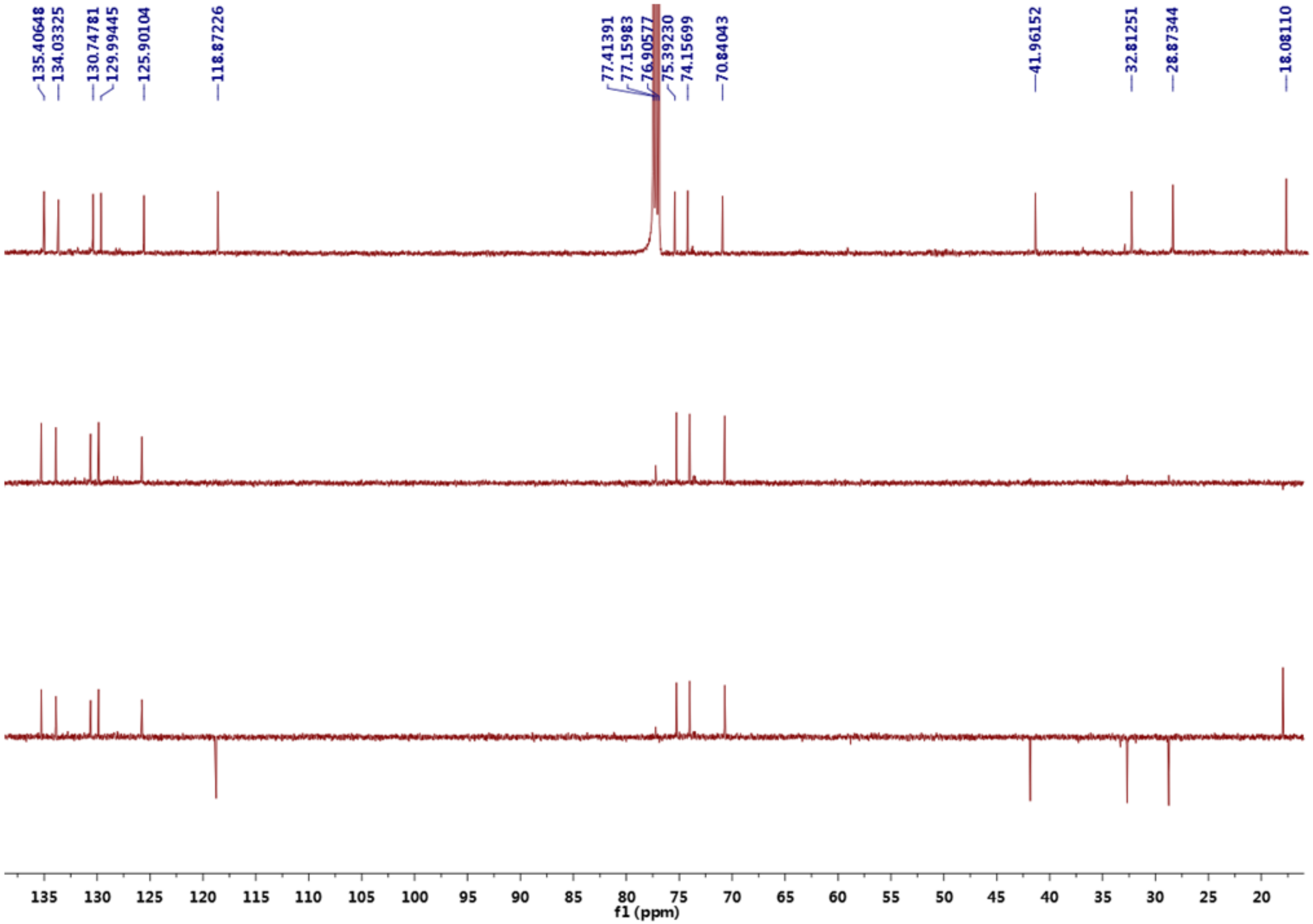

Figure S16. ^{13}C NMR and DEPT spectra of compound **3** in $CDCl_3$

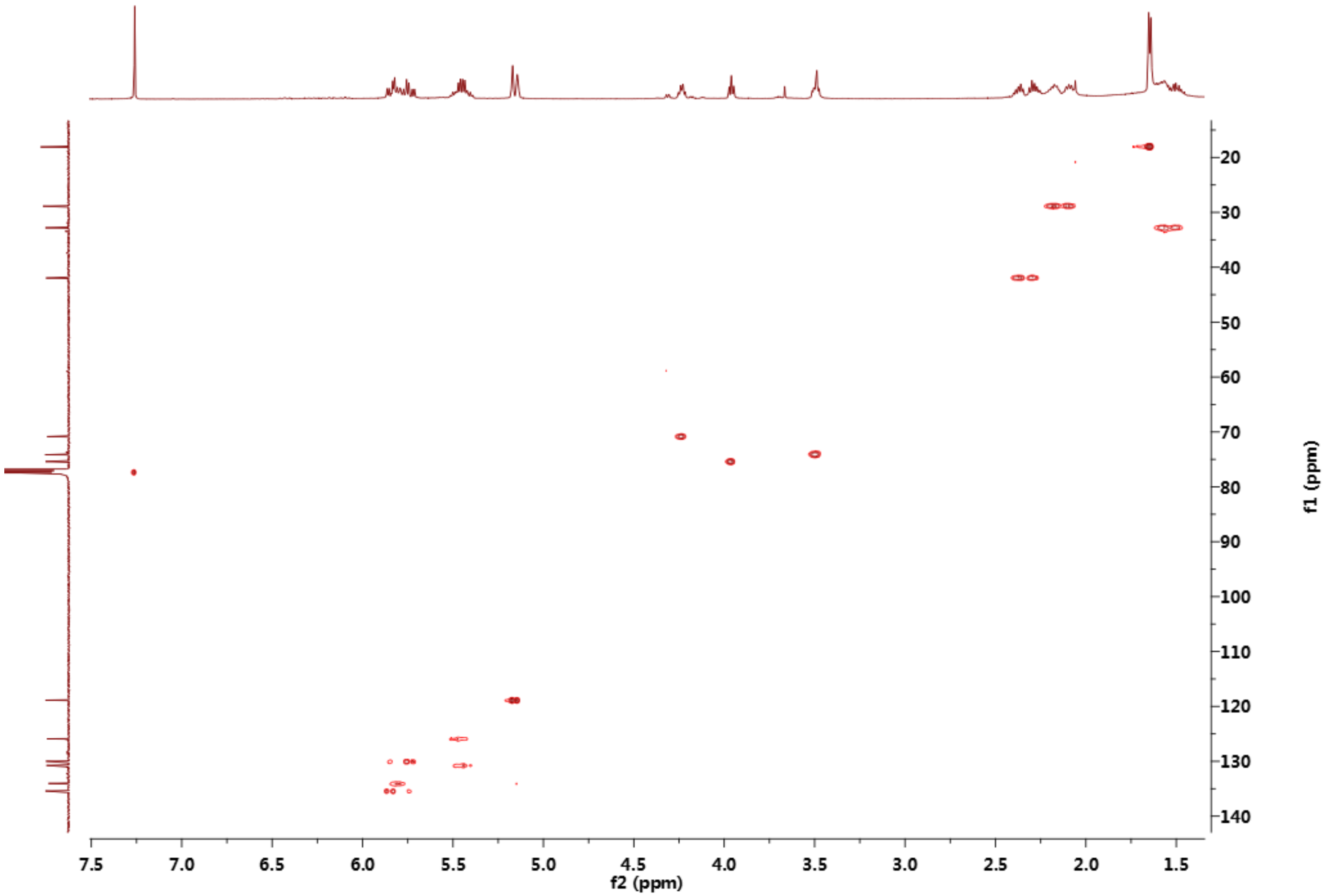

Figure S17. HSQC spectrum of compound **3** in $CDCl_3$

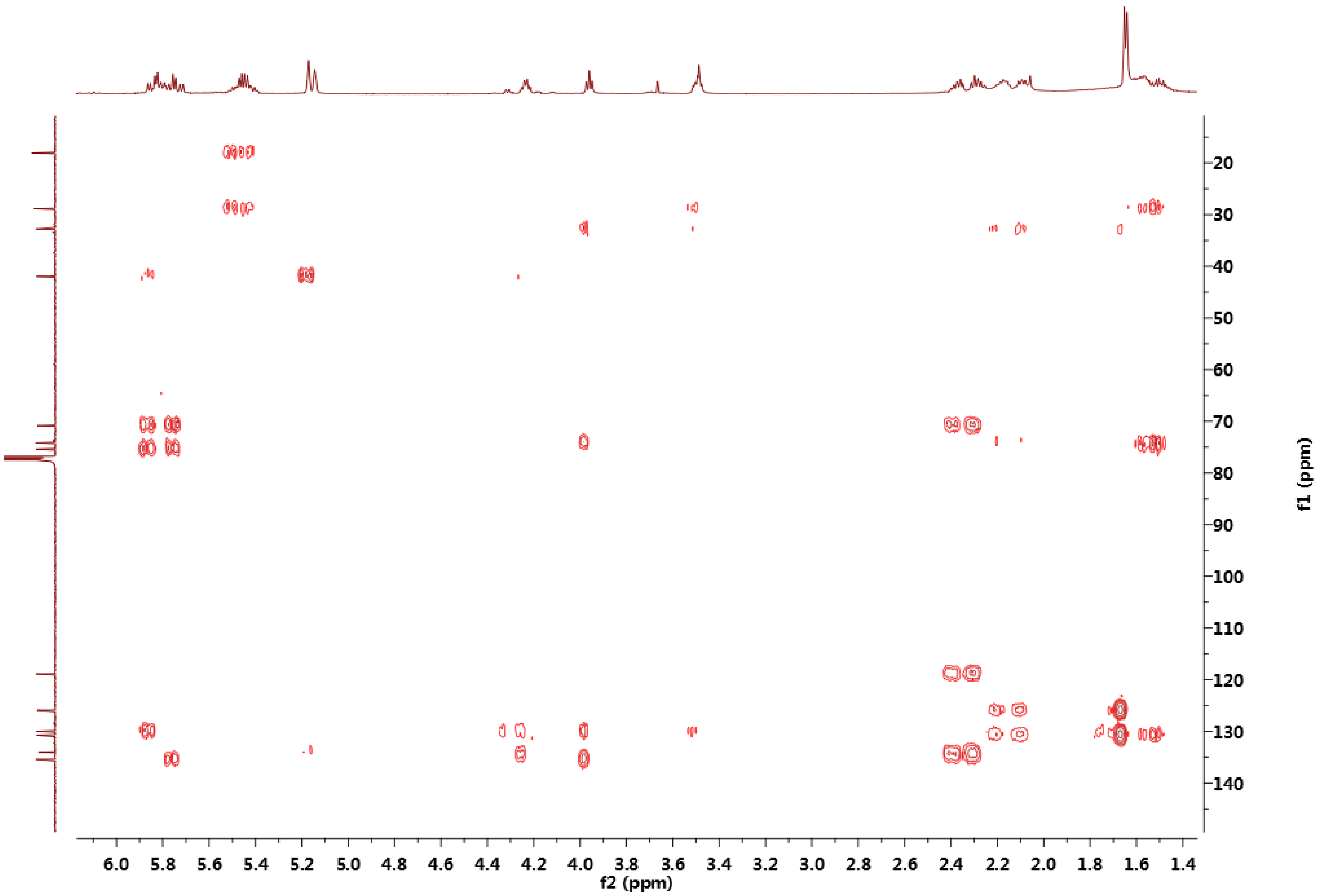

Figure S18. HMBC spectrum of compound **3** in $CDCl_3$

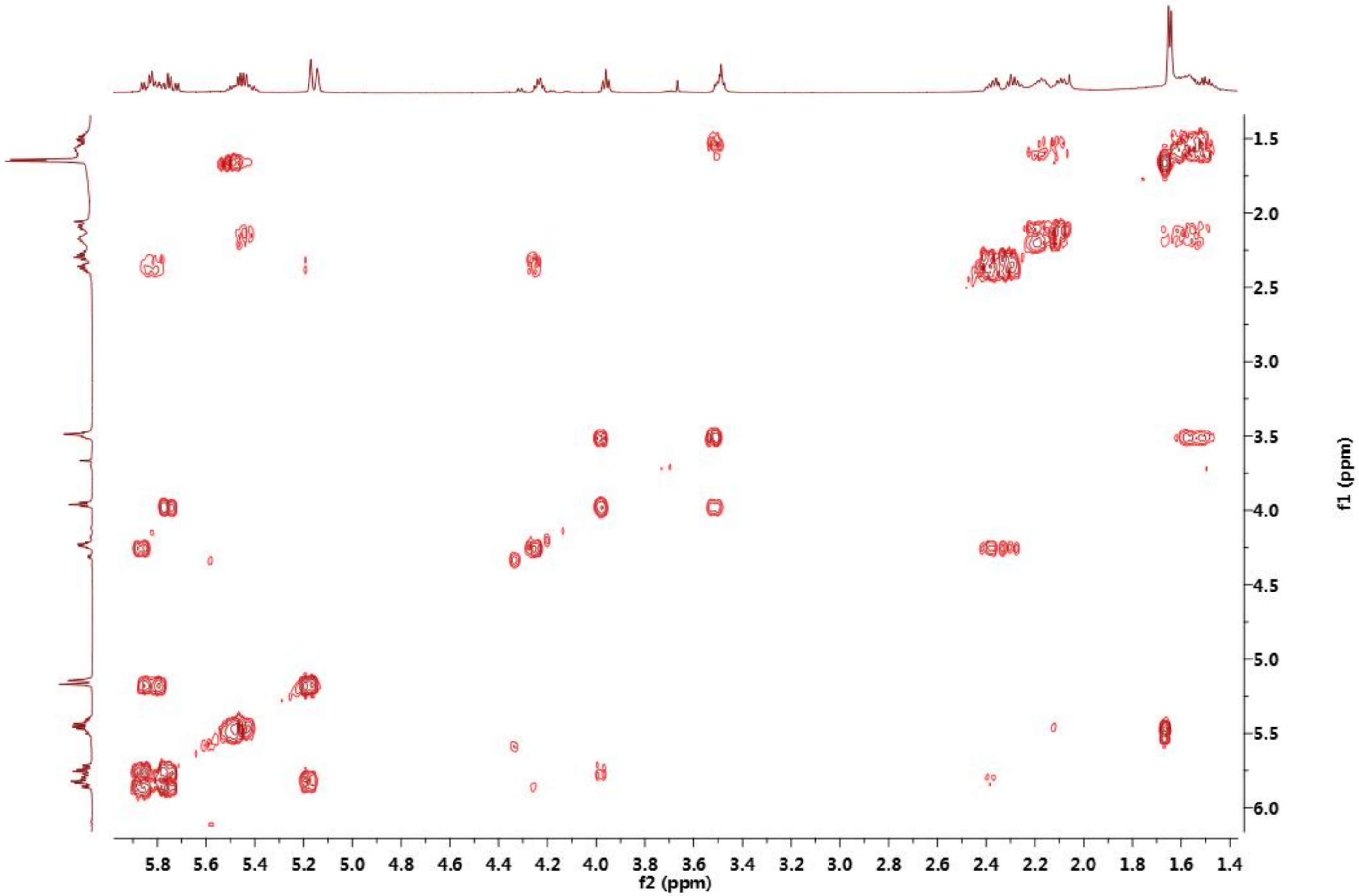

Figure S19. COSY spectrum of compound **3** in $CDCl_3$

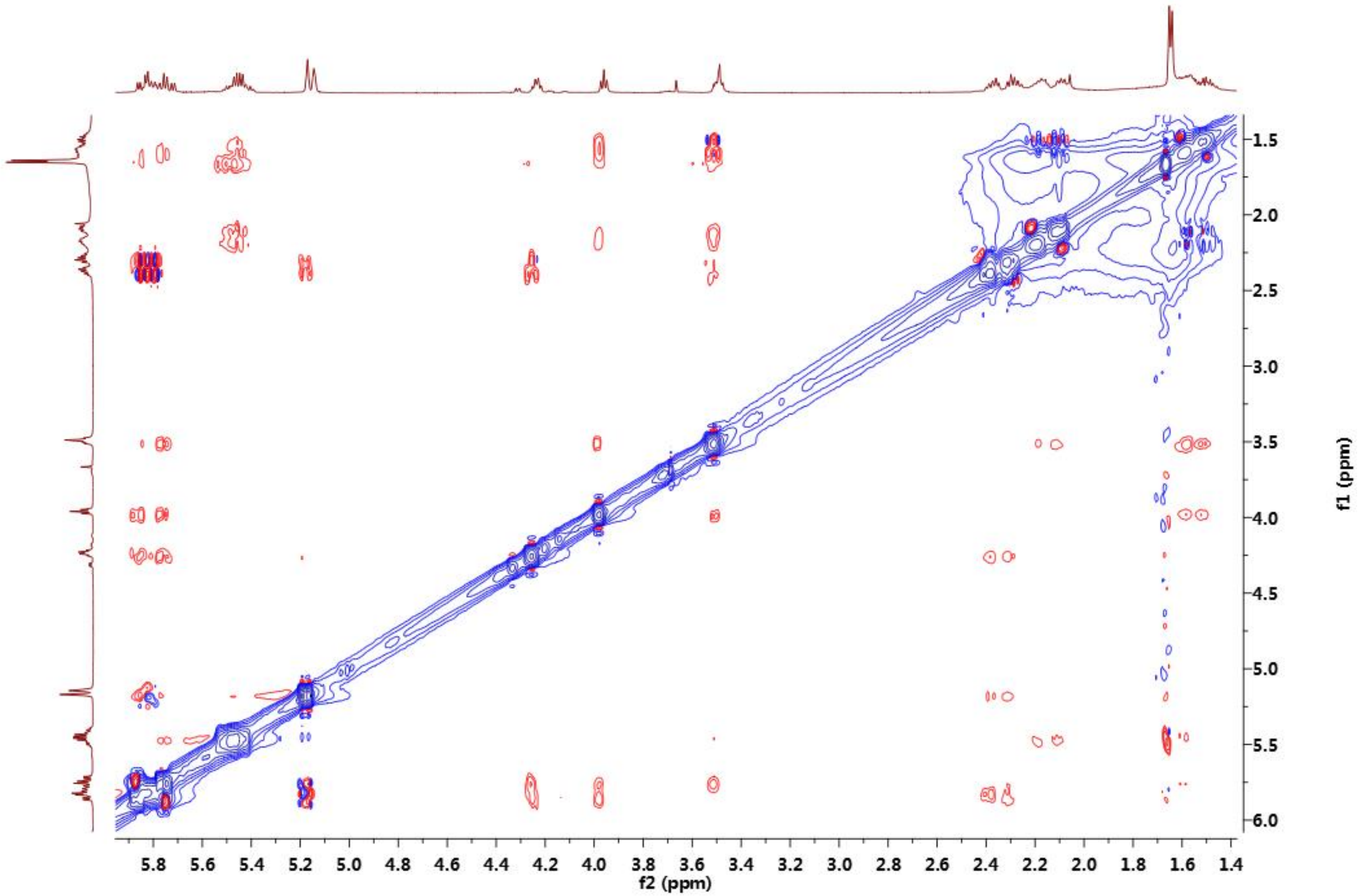

Figure S20. NOESY spectrum of compound **3** in $CDCl_3$

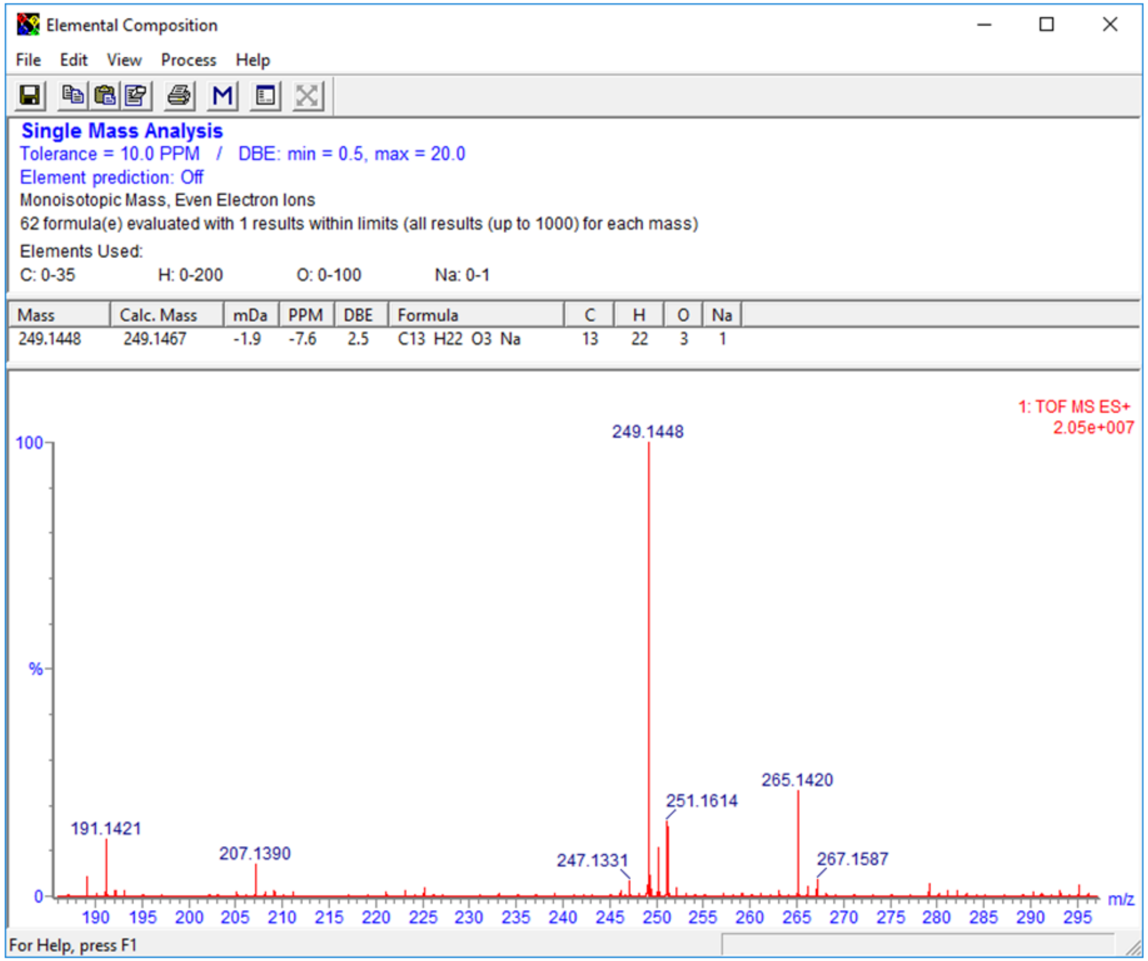

Figure S21. HRESIMS spectrum of compound **3**

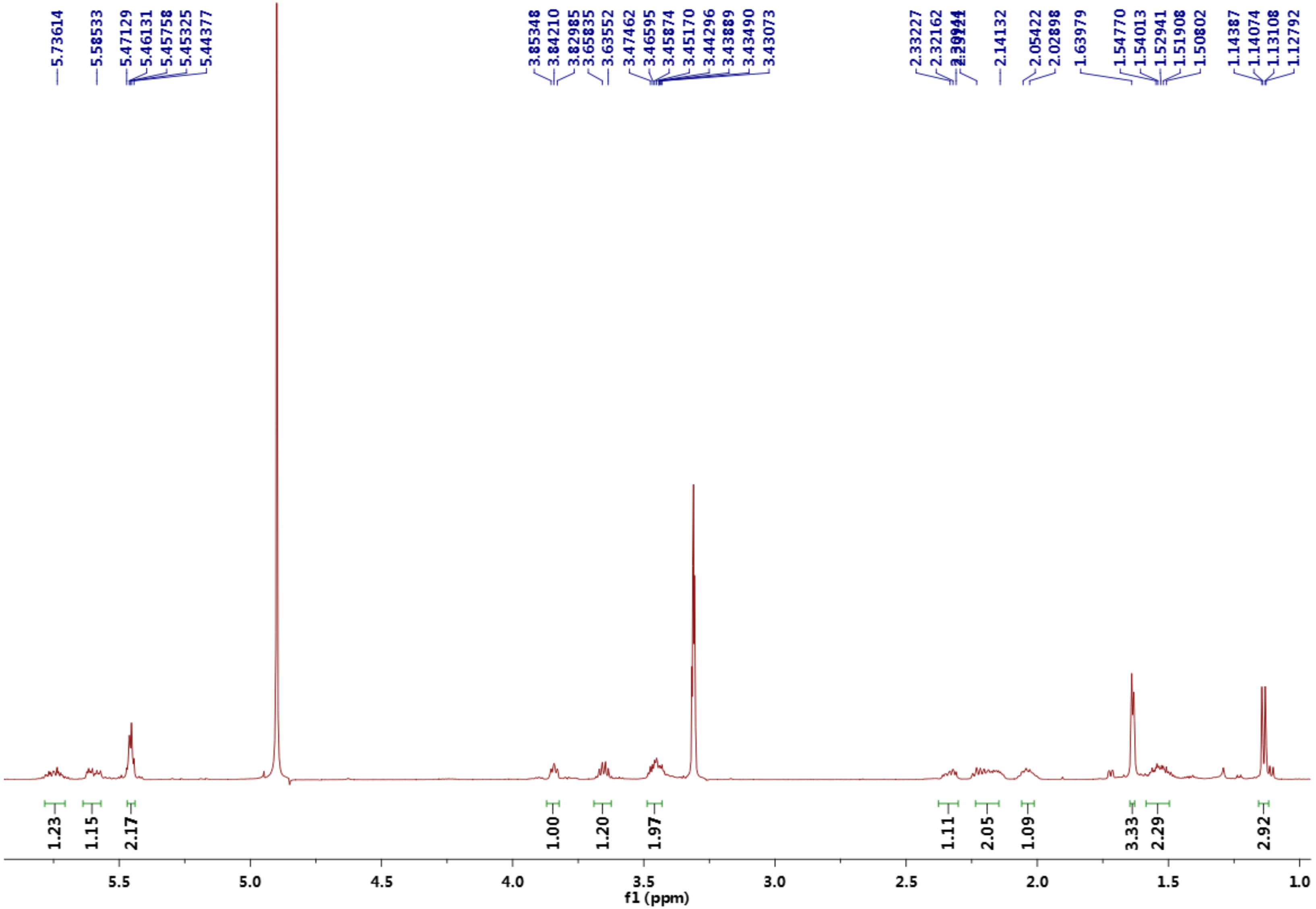

Figure S22. ^{1}H NMR spectrum of compound **4** in CD_3OD

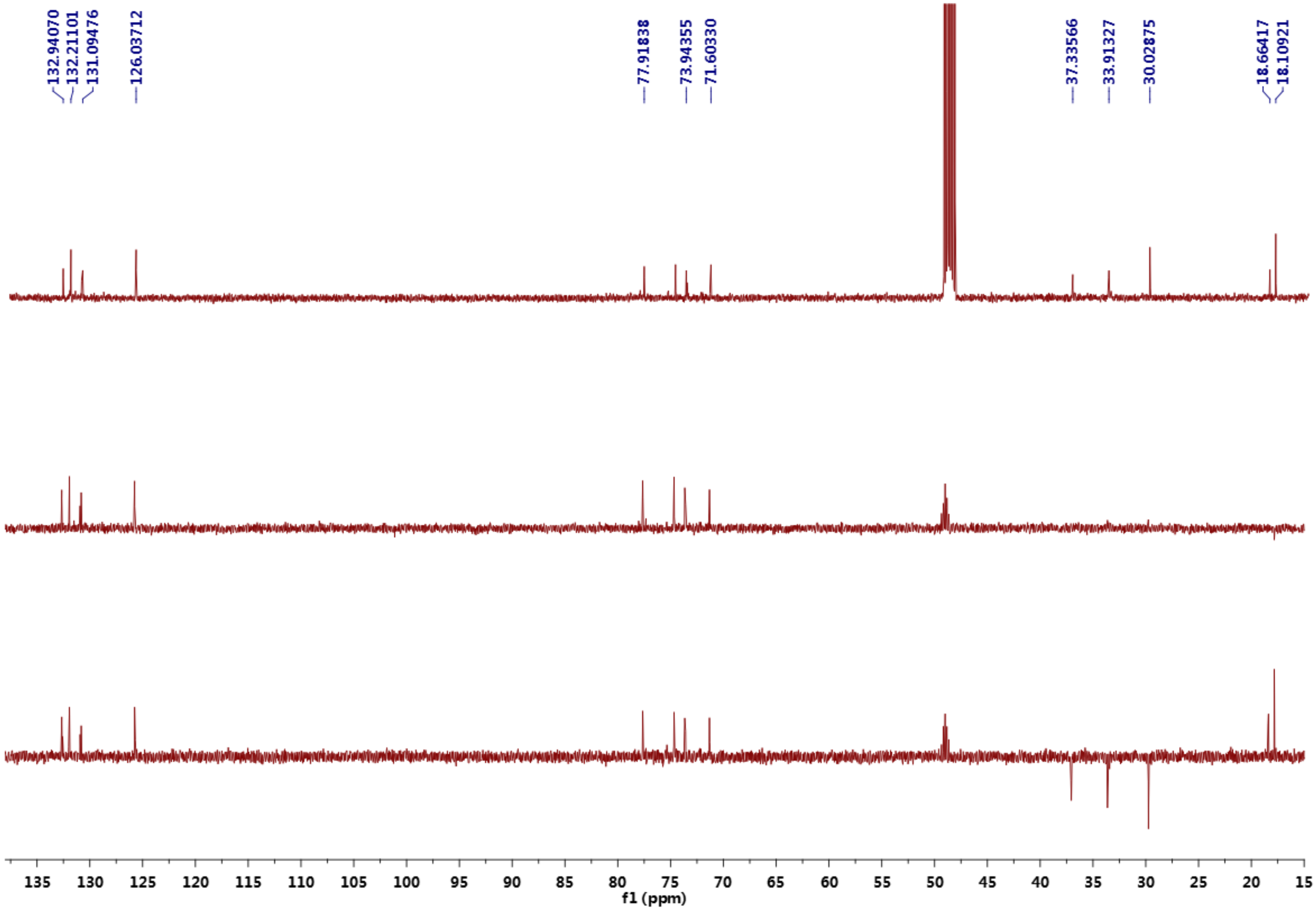

Figure S23. ^{13}C NMR and DEPT spectra of compound **4** in CD_3OD

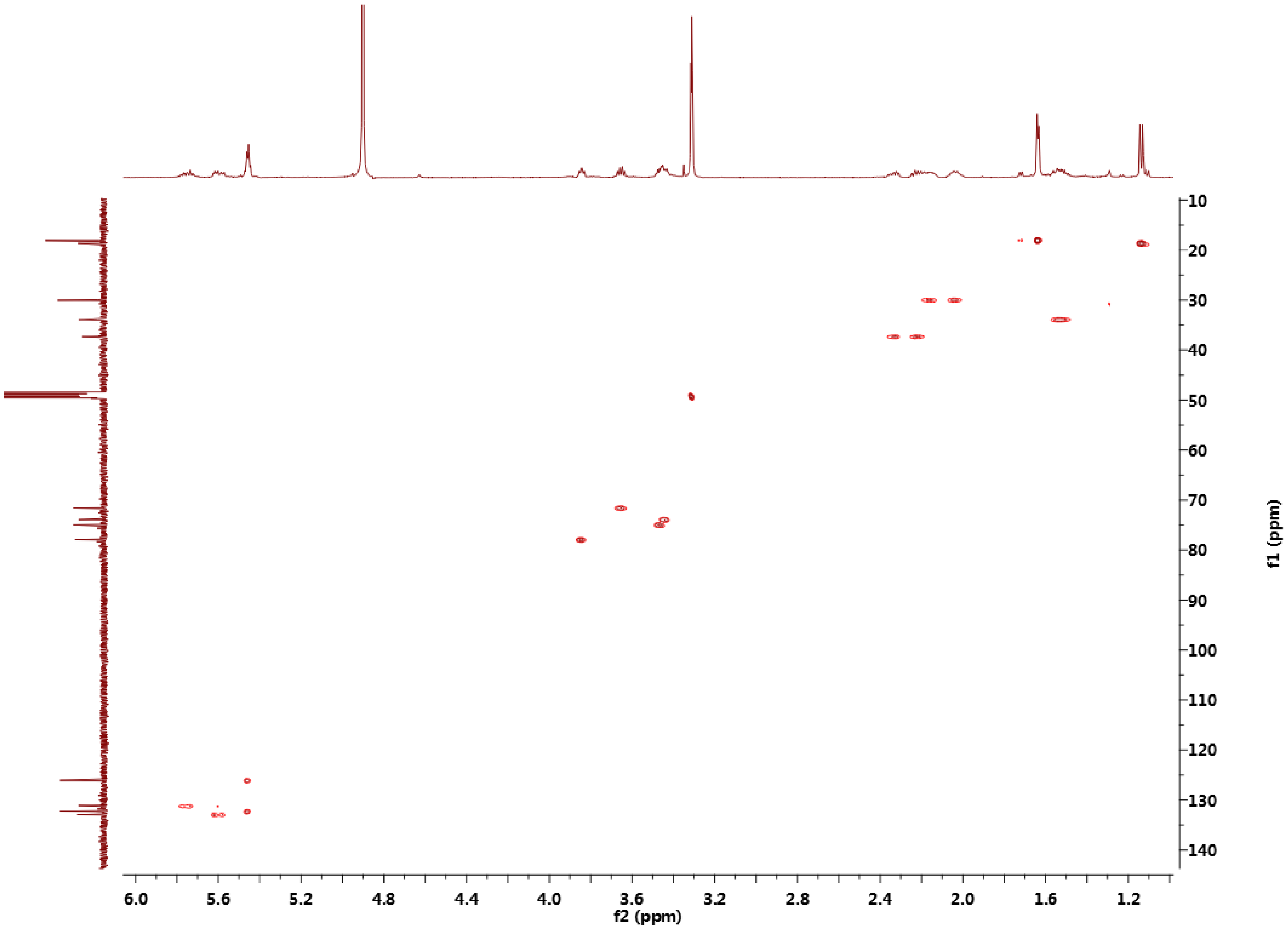

Figure S24. HSQC spectrum of compound **4** in CD_3OD

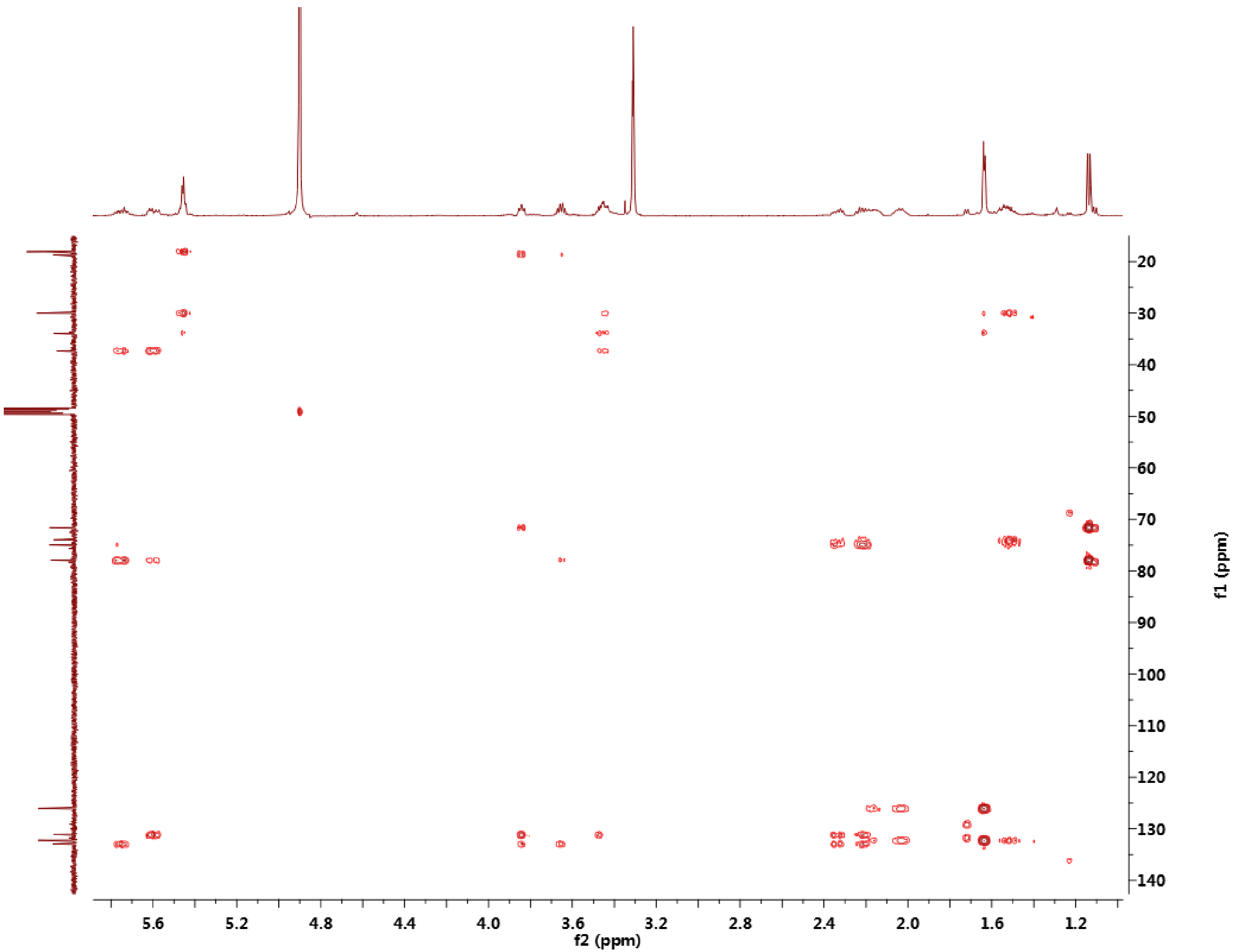

Figure S25. HMBC spectrum of compound **4** in CD_3OD

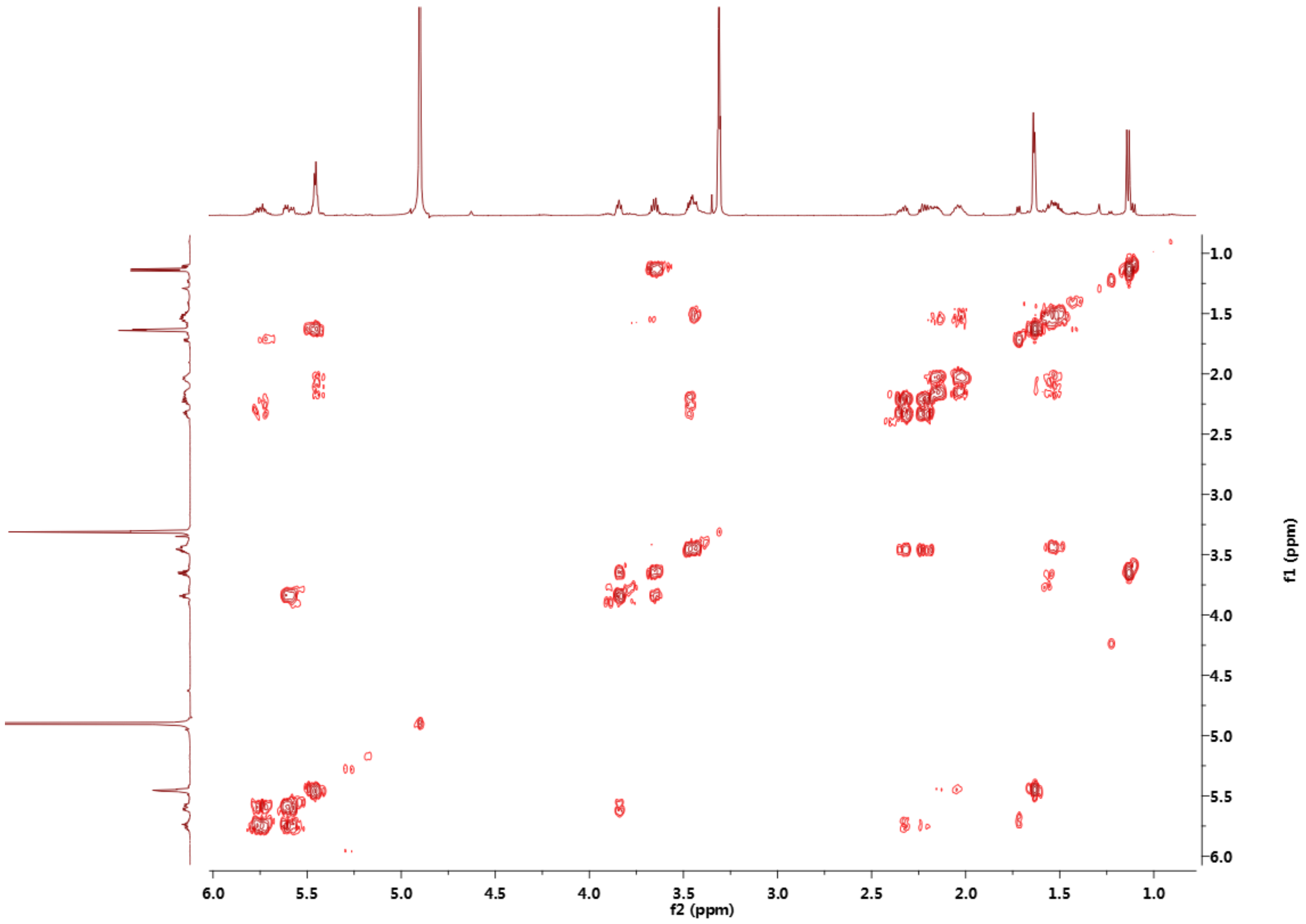

Figure S26. COSY spectrum of compound **4** in CD_3OD

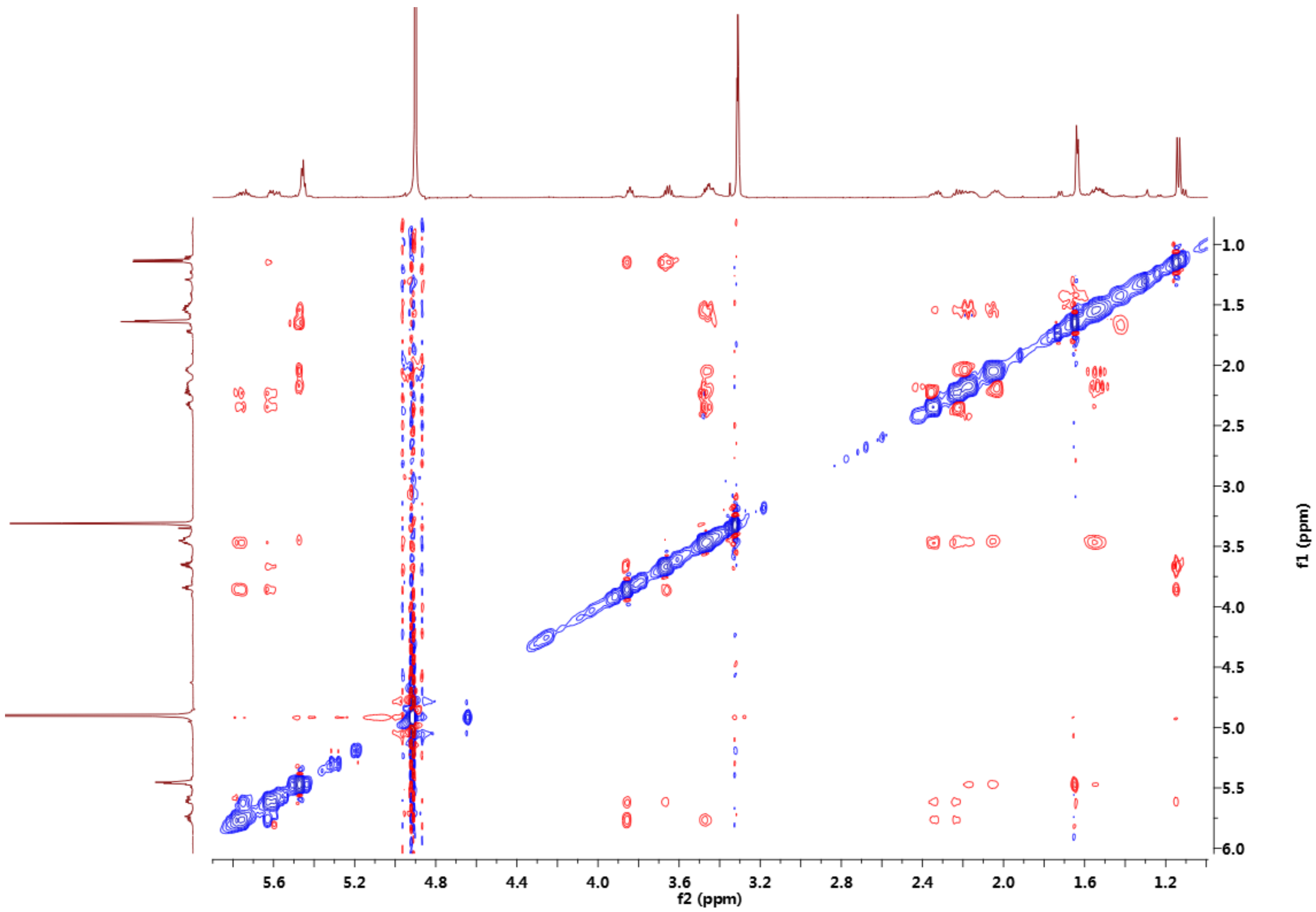

Figure S27. NOESY spectrum of compound **4** in CD_3OD

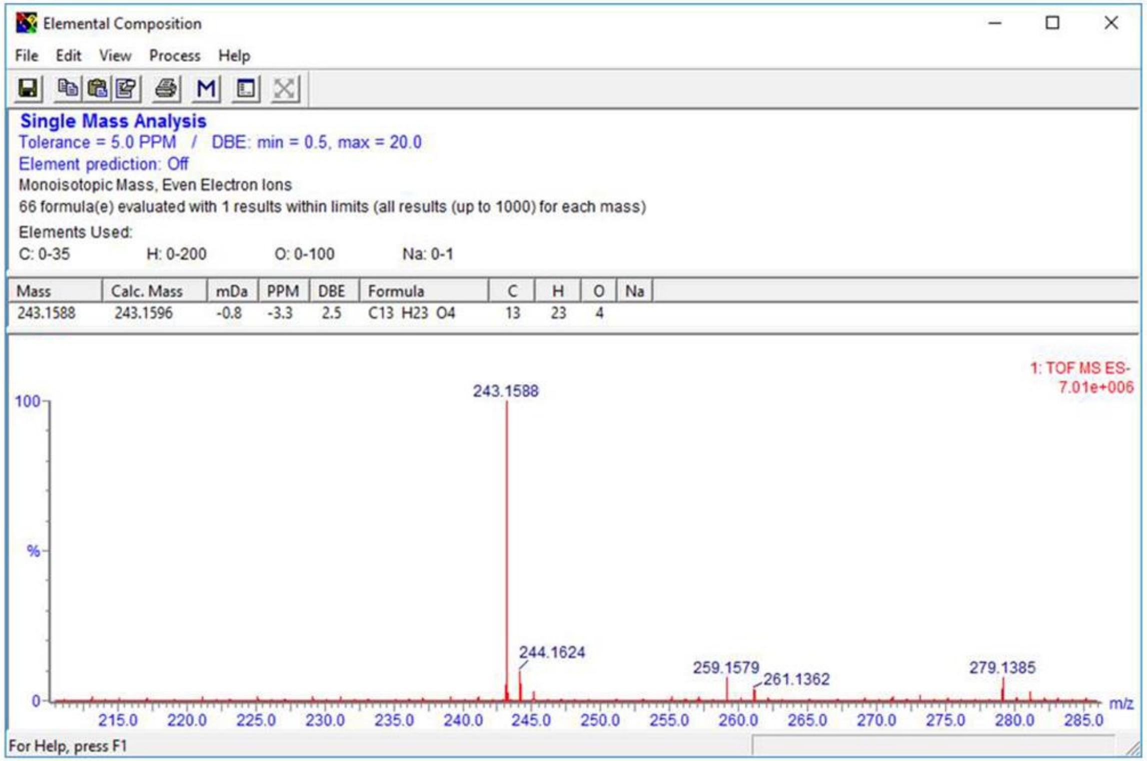

Figure S28. HRESIMS spectrum of compound **4**

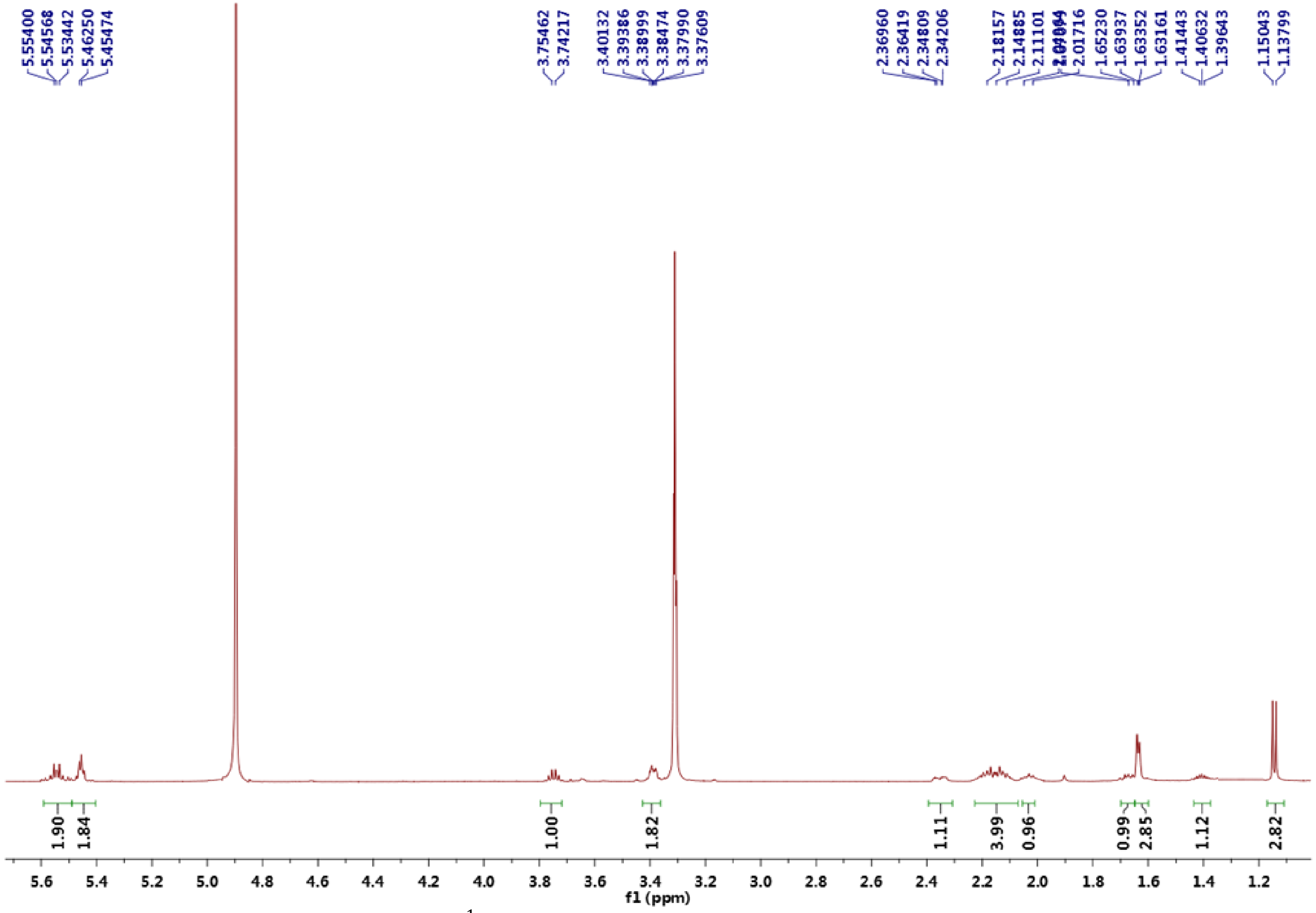

Figure S29. ^{1}H NMR spectrum of compound **5** in CD_3OD

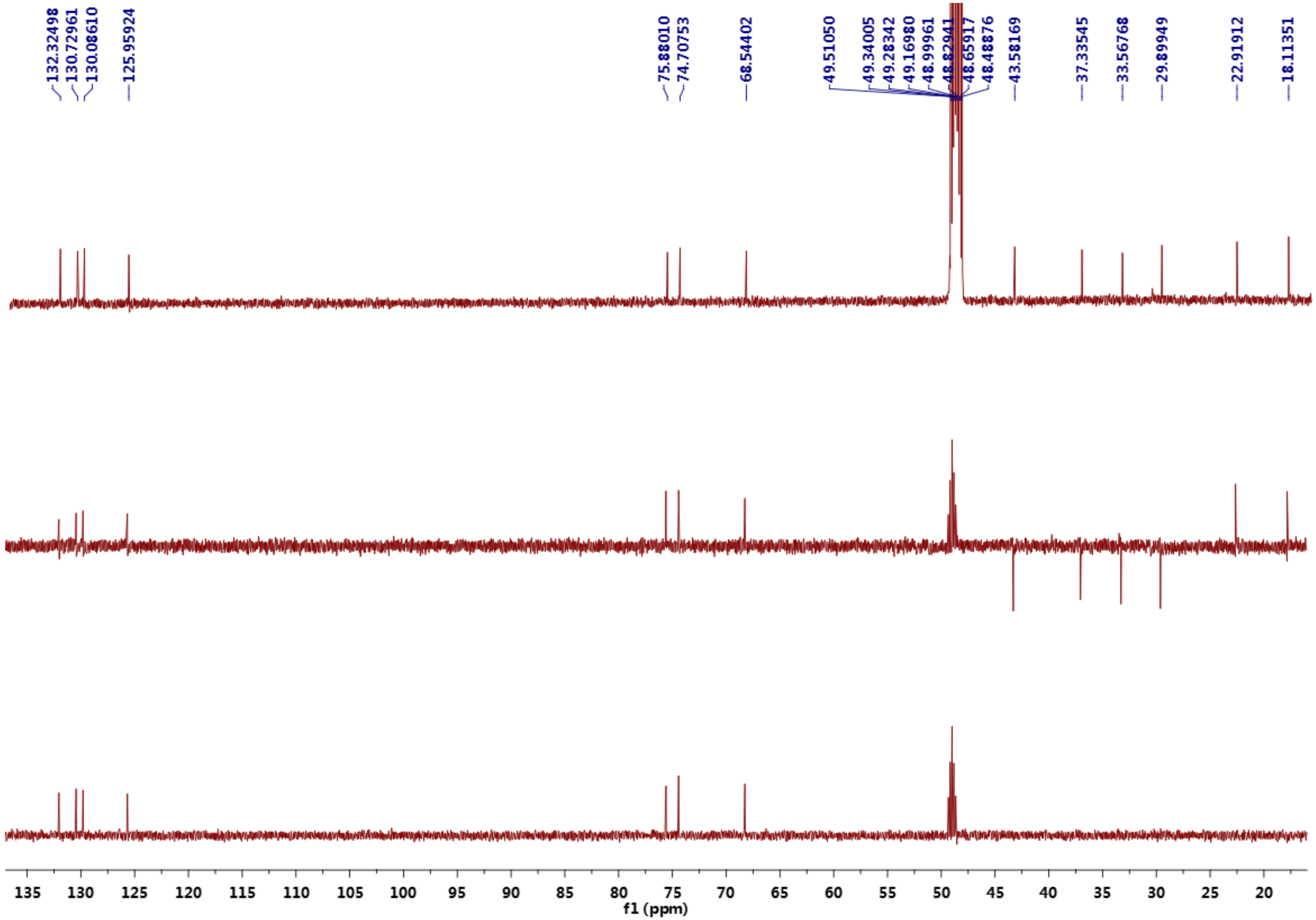

Figure S30. ^{13}C NMR and DEPT spectra of compound **5** in CD_3OD

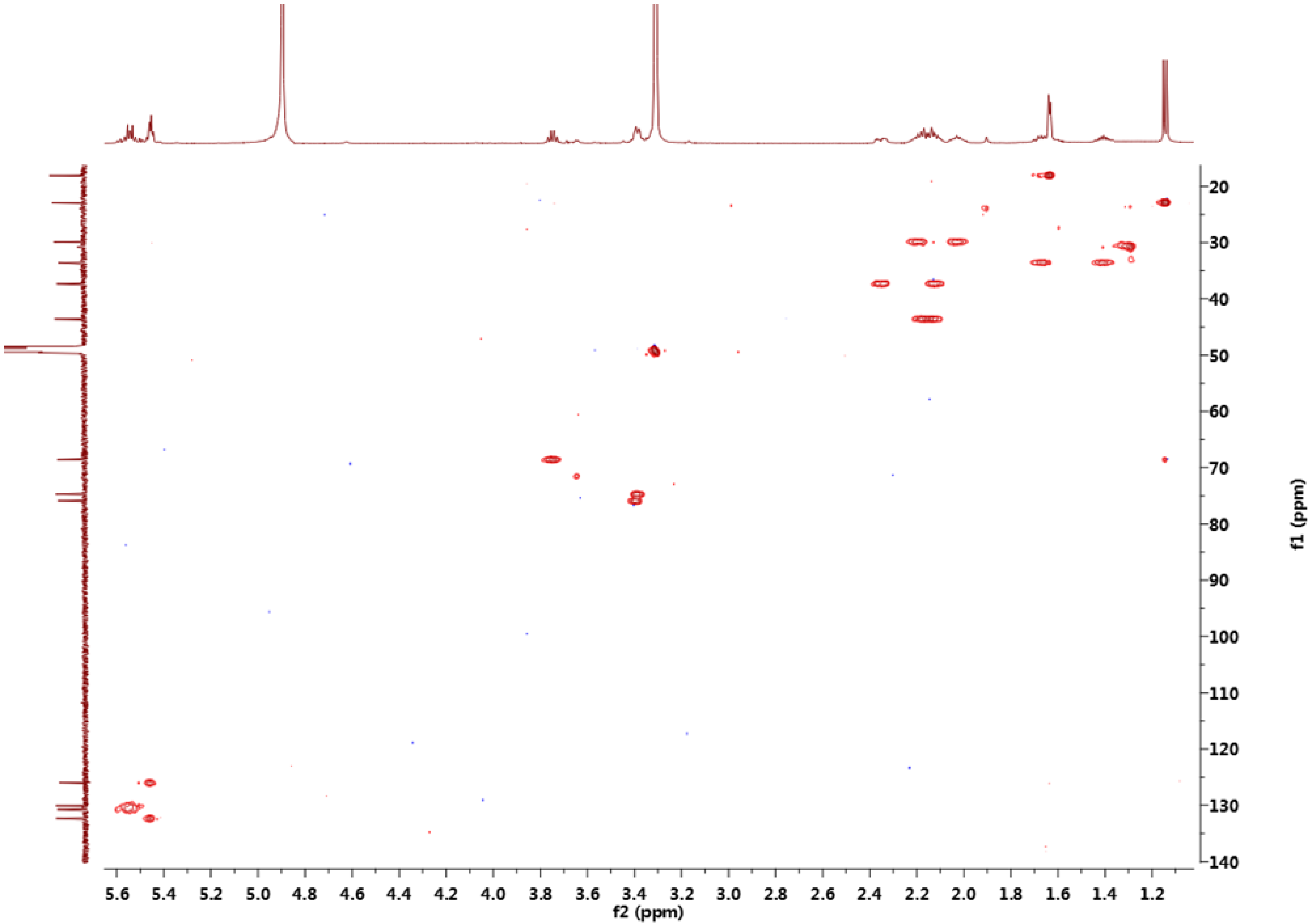

Figure S31. HSQC spectrum of compound **5** in CD_3OD

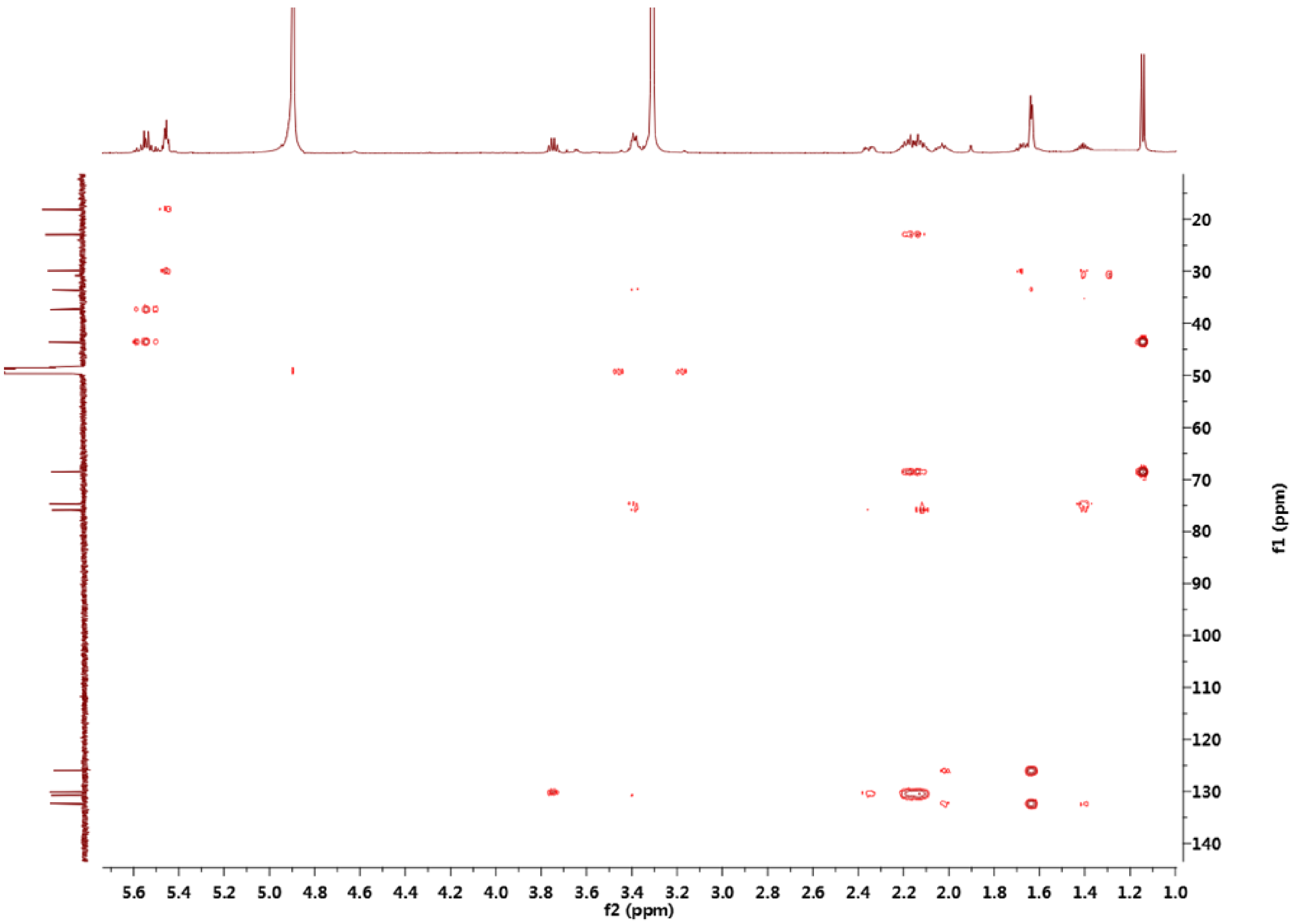

Figure S32. HMBC spectrum of compound **5** in CD_3OD

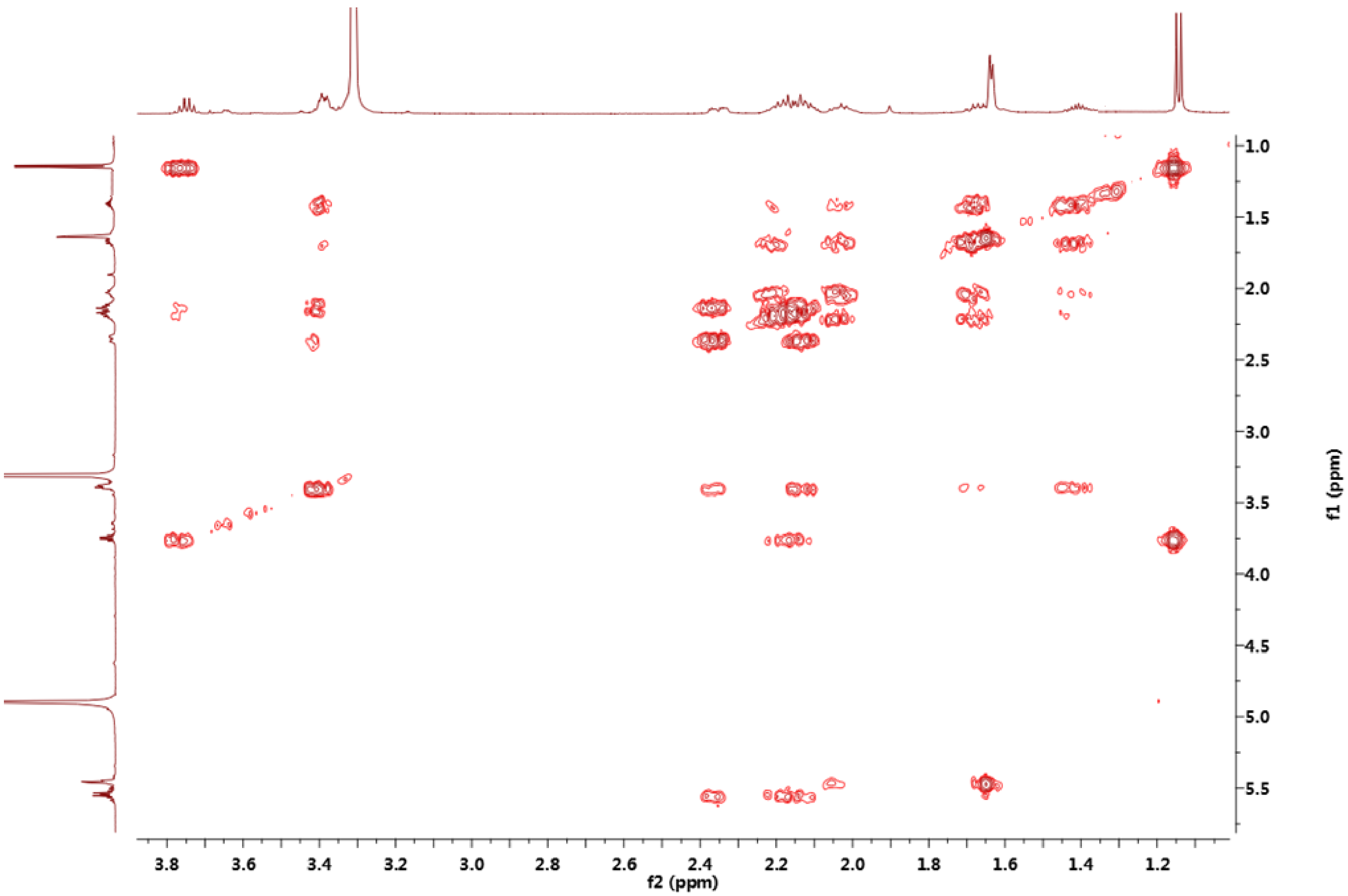

Figure S33. COSY spectrum of compound **5** in CD_3OD

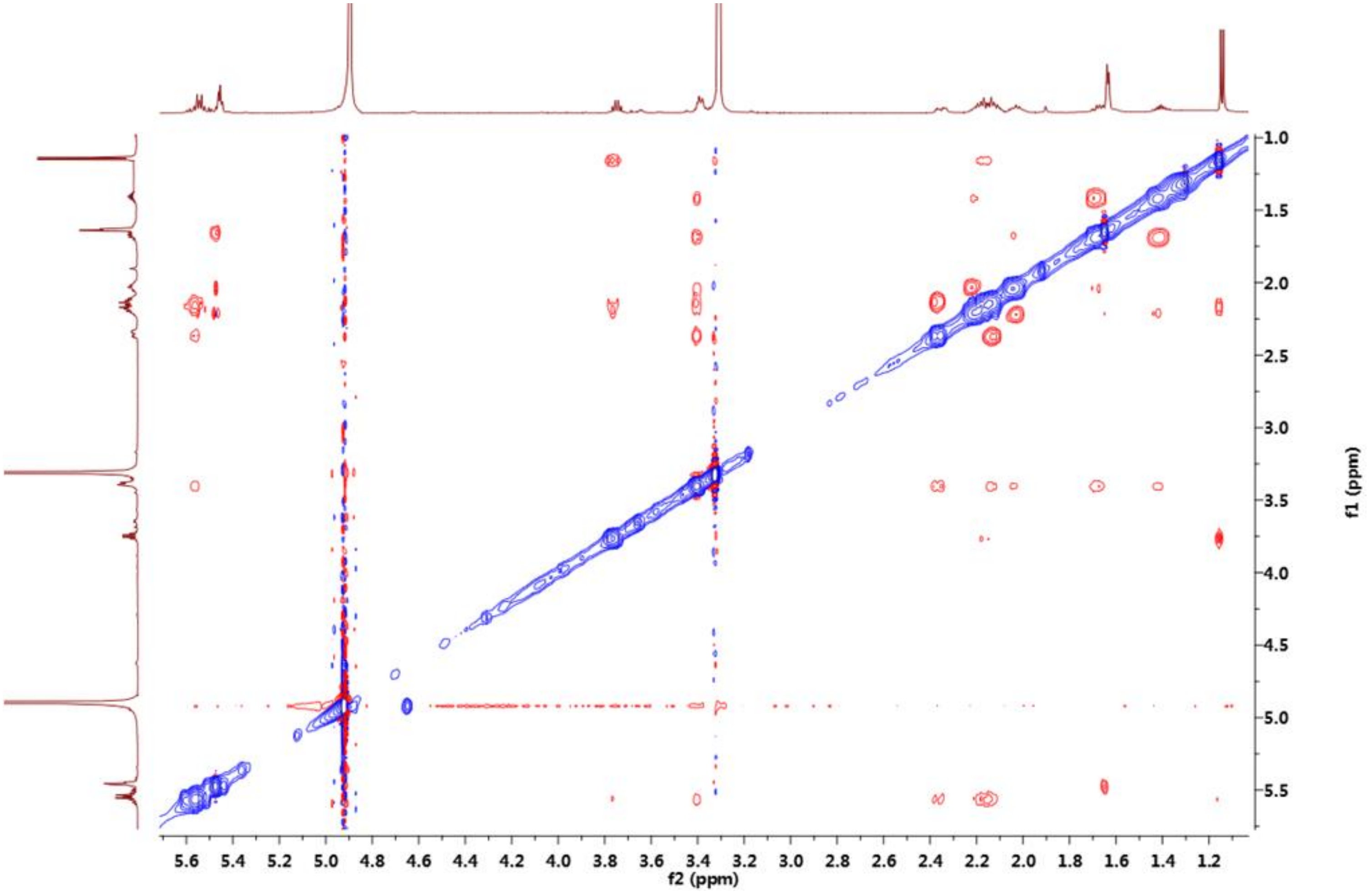

Figure S34. NOESY spectrum of compound **5** in CD_3OD

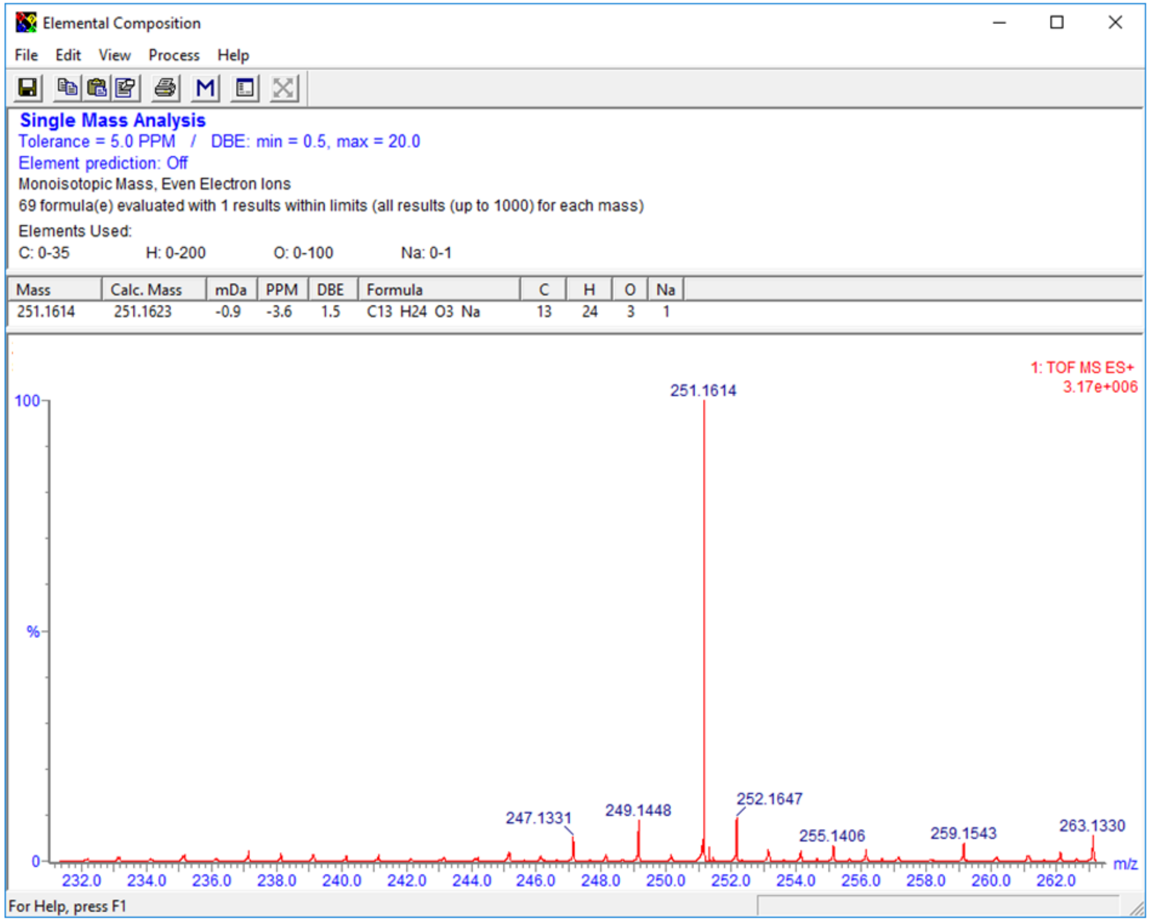

Figure S35. HRESIMS spectrum of compound **5**

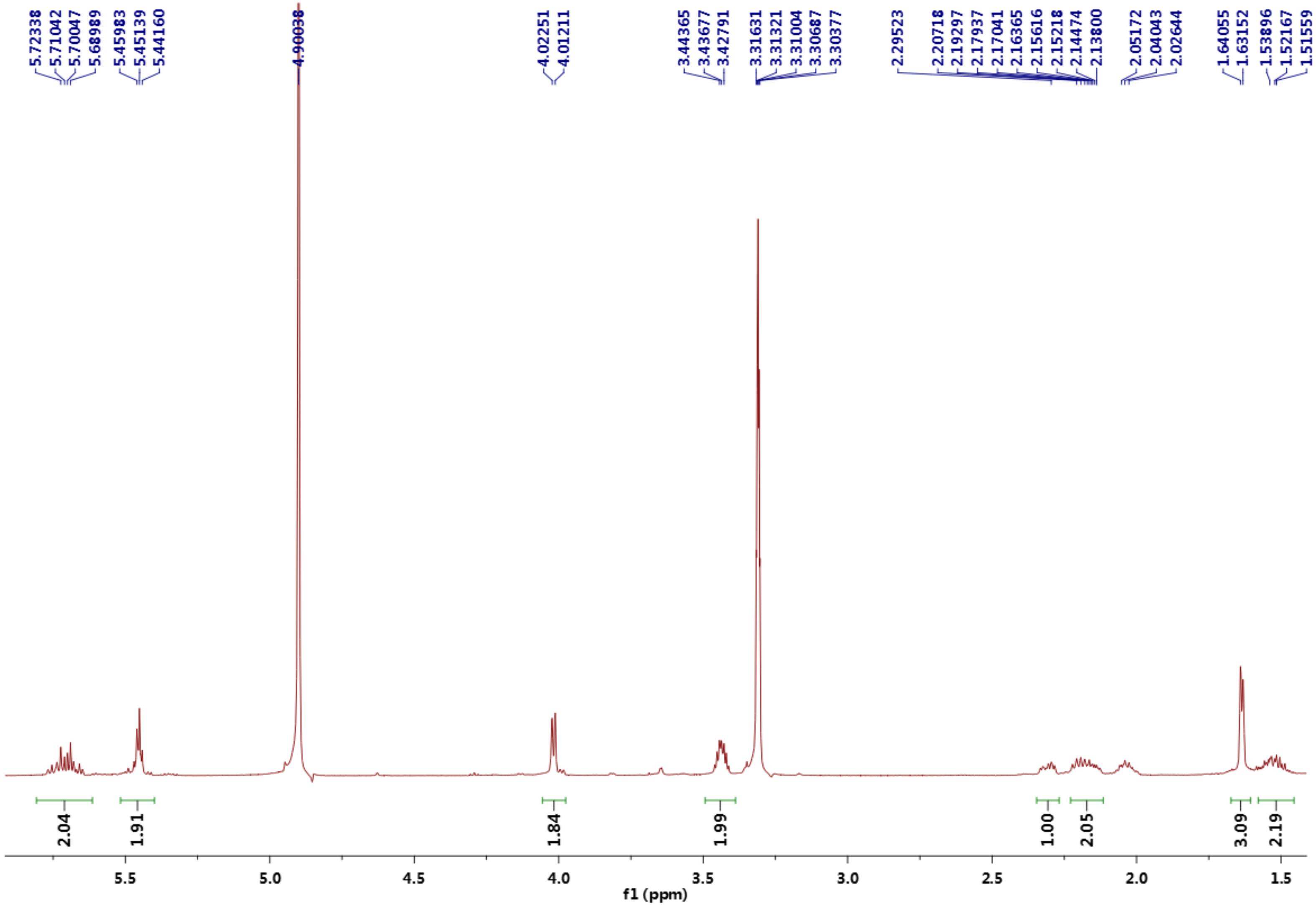

Figure S36. ^{1}H NMR spectrum of compound **6** in CD_3OD

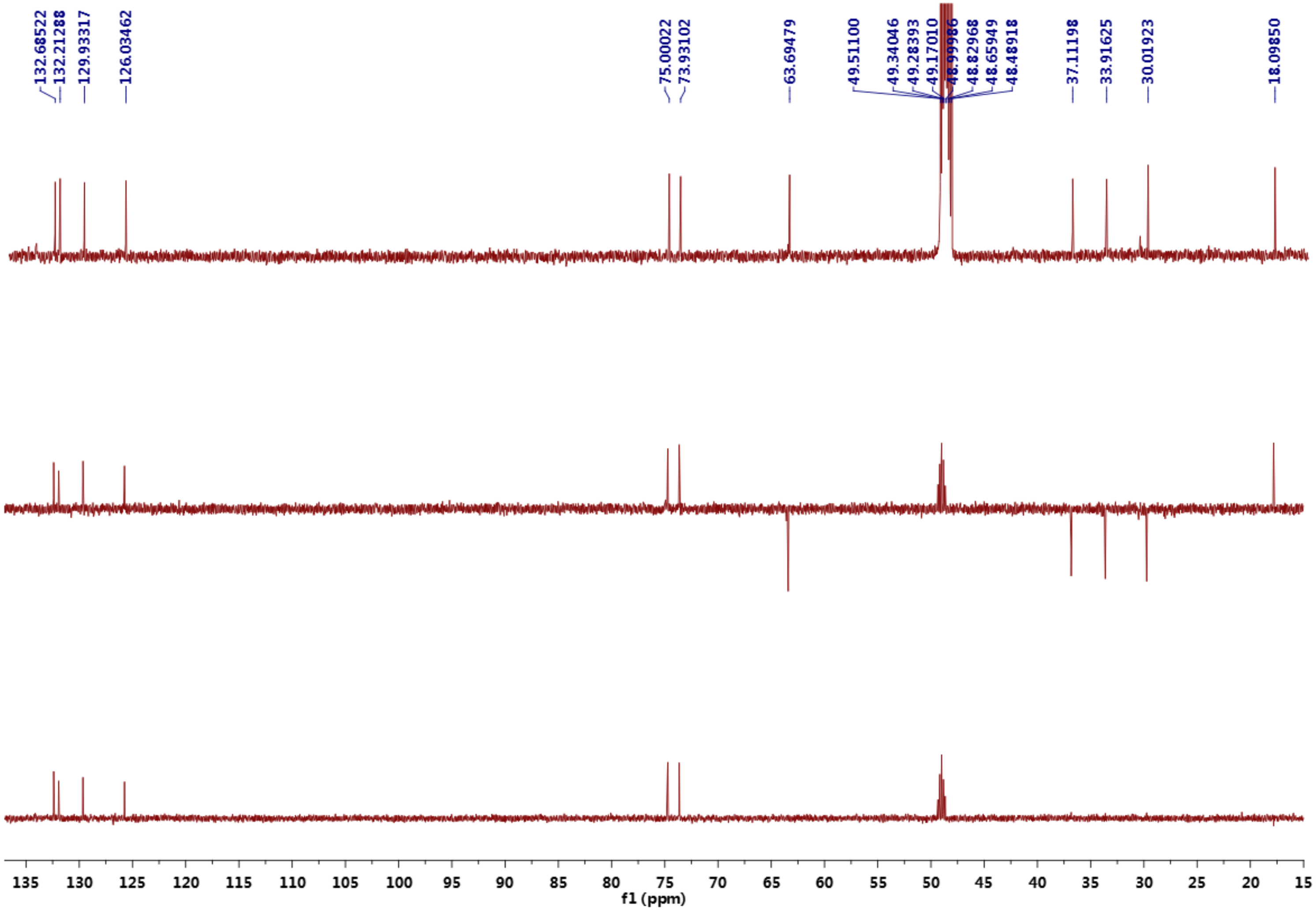

Figure S37. ^{13}C NMR and DEPT spectra of compound **6** in CD_3OD

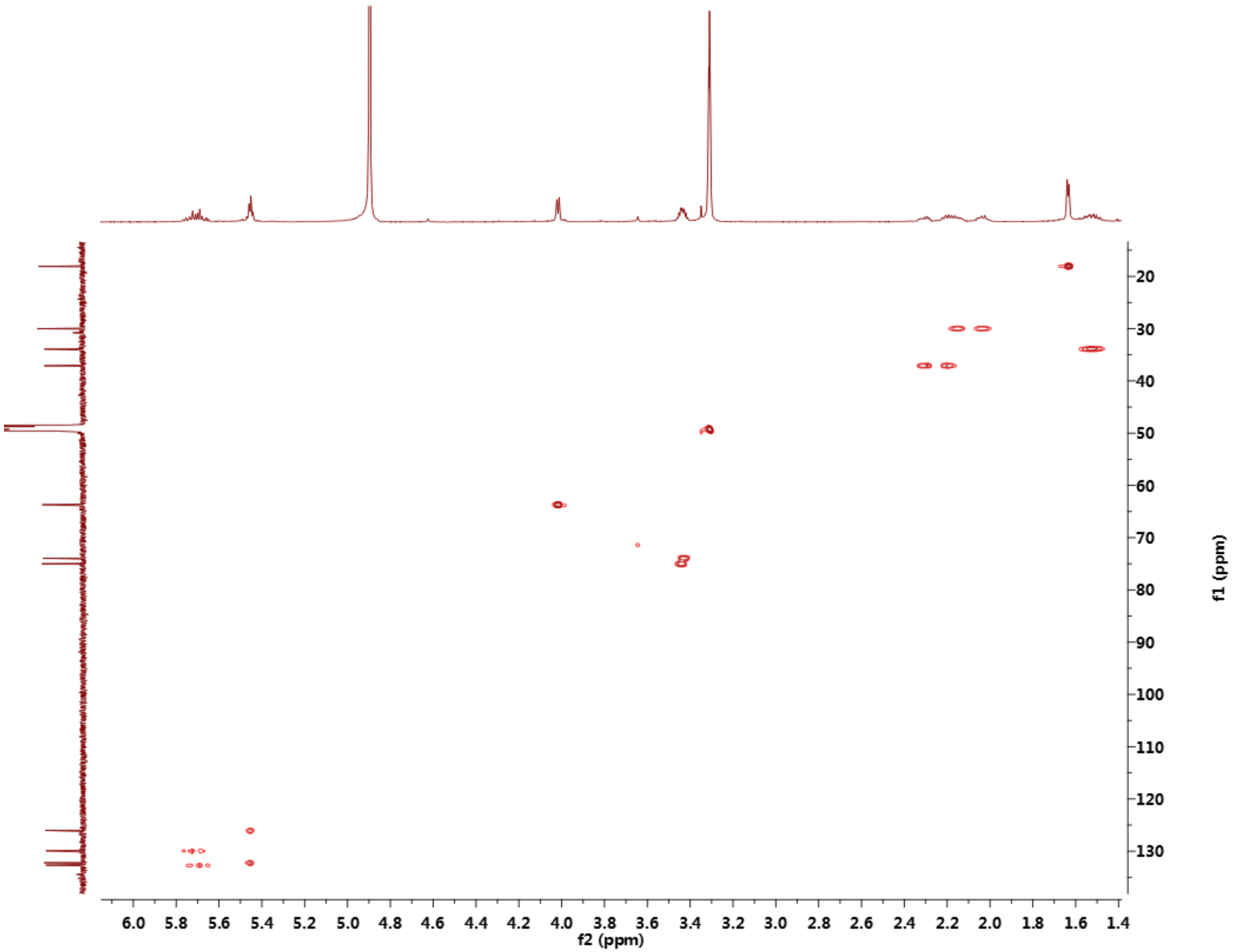

Figure S38. HSQC spectrum of compound **6** in CD_3OD

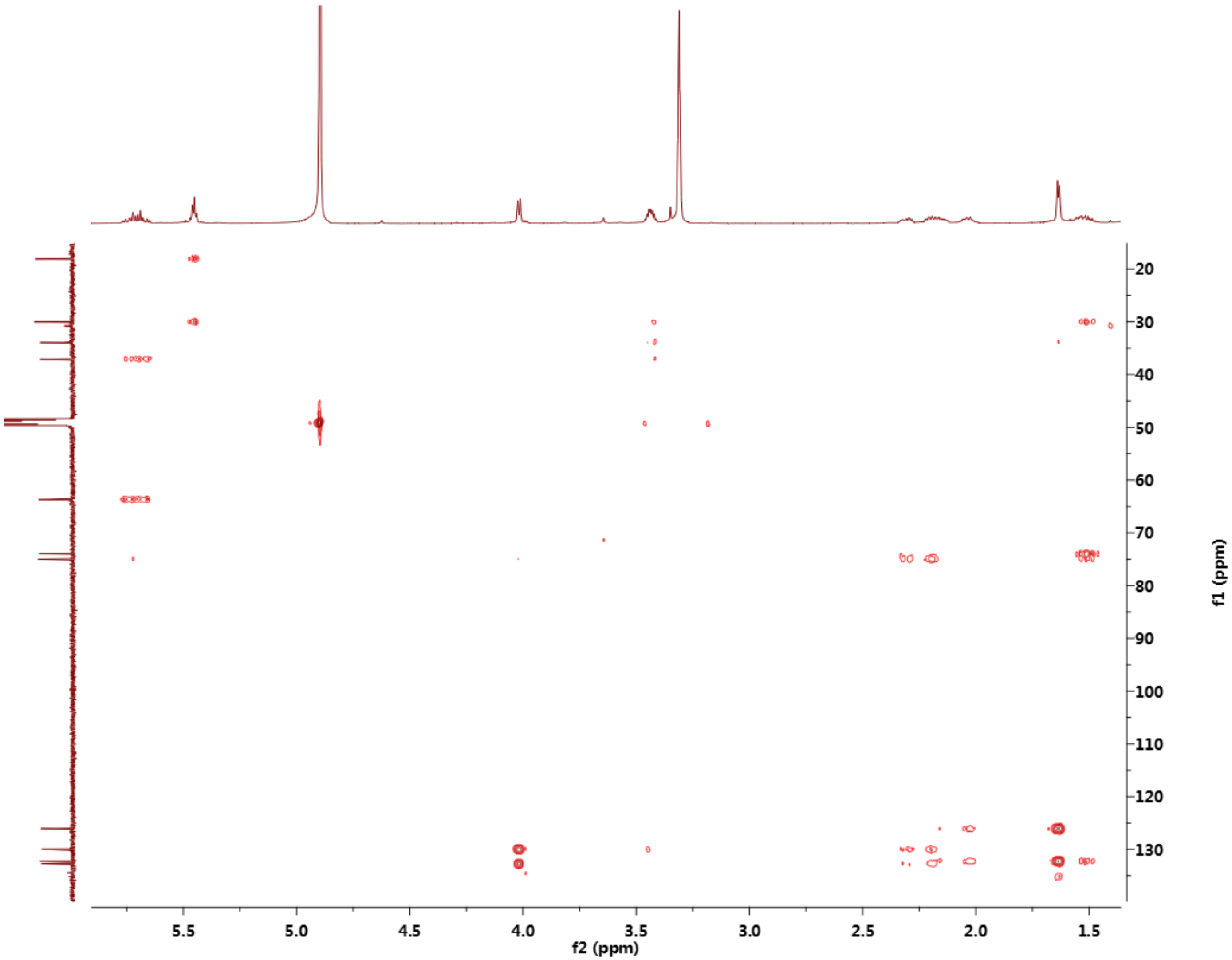

Figure S39. HMBC spectrum of compound **6** in CD_3OD

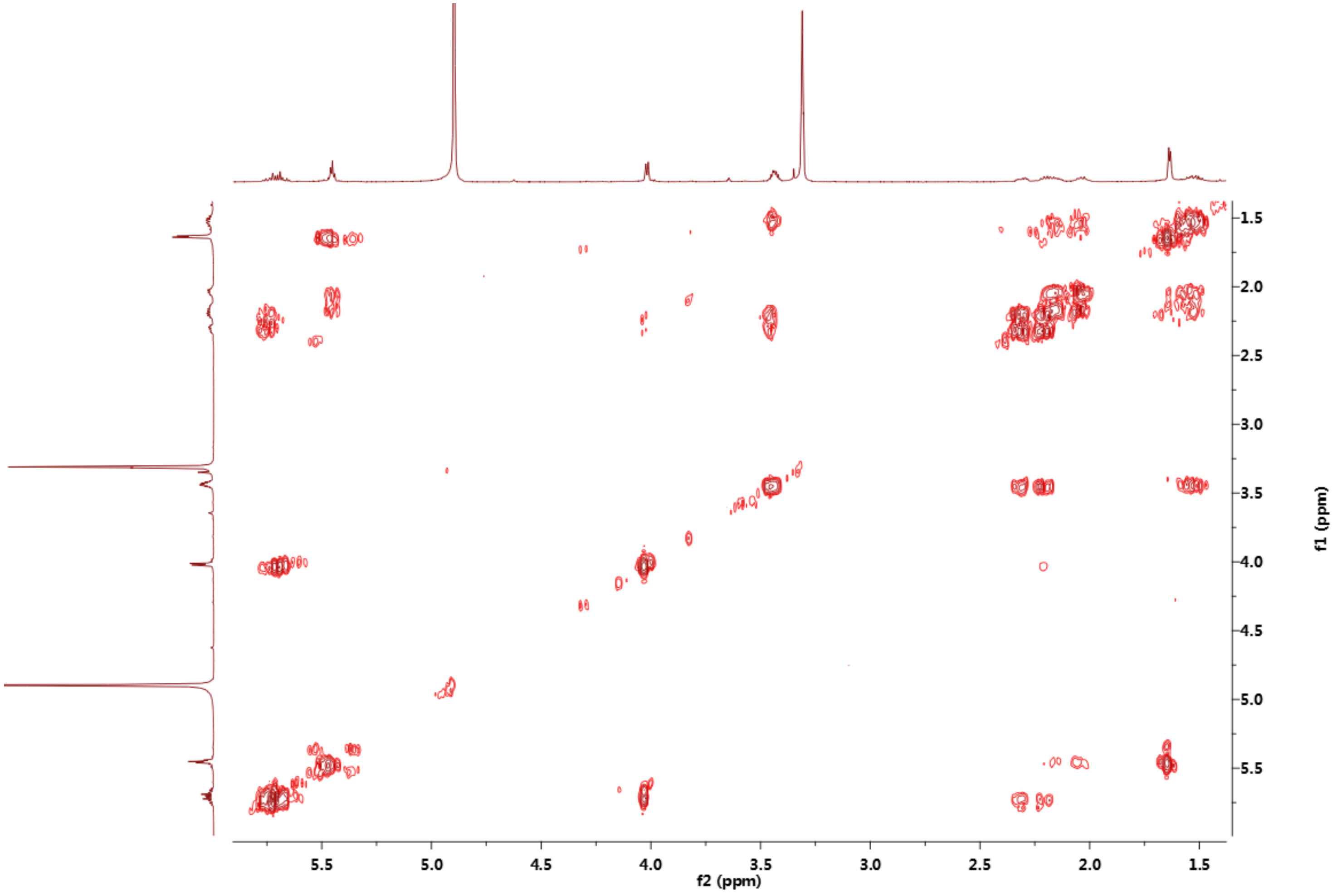

Figure S40. COSY spectrum of compound **6** in CD_3OD

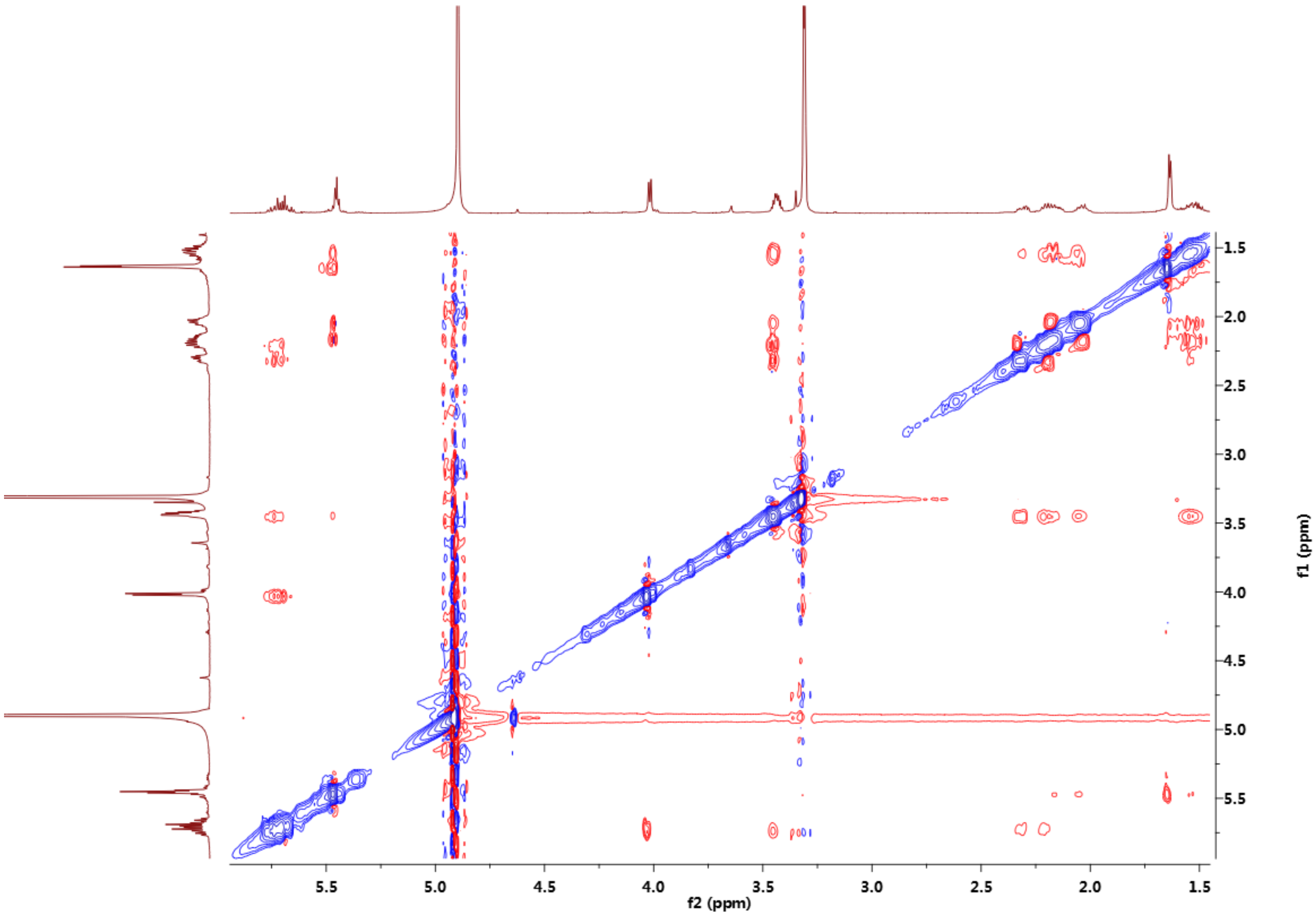

Figure S41. NOESY spectrum of compound **6** in CD_3OD

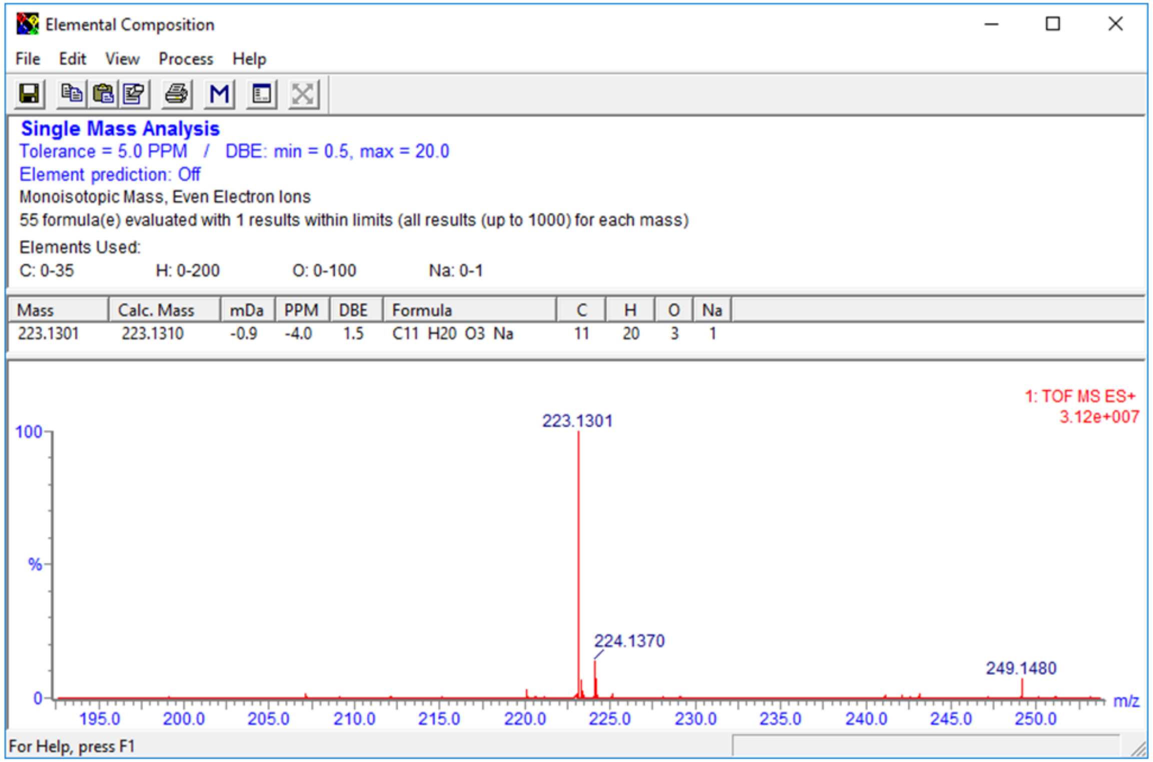

Figure S42. HRESIMS spectrum of compound **6**
